# Boron Neutron Capture Therapy‐Derived Extracellular Vesicles via DNA Accumulation Boost Antitumor Dendritic Cell Vaccine Efficacy

**DOI:** 10.1002/advs.202405158

**Published:** 2024-07-17

**Authors:** Linwen Lv, Junzhe Zhang, Yujiao Wang, Haojun Liang, Qiuyang Liu, Fan Hu, Hao Li, Wenxi Su, Junhui Zhang, Ranran Chen, Ziteng Chen, Zhijie Wang, Jiacheng Li, Ruyu Yan, Mingxin Yang, Ya‐nan Chang, Juan Li, Tianjiao Liang, Gengmei Xing, Kui Chen

**Affiliations:** ^1^ CAS Key Lab for Biomedical Effects of Nanomaterials and Nanosafety Institute of High Energy Physics Chinese Academy of Sciences 19B YuquanLu, Shijingshan District Beijing 100049 China; ^2^ University of Chinese Academy of Sciences Beijing 100049 China; ^3^ State Key Laboratory for Quality Ensurance and Sustainable Use of Dao‐di Herbs Artemisinin Research Center and Institute of Chinese Materia Medica China Academy of Chinese Medical Sciences Beijing 100700 China; ^4^ Guangdong‐Hong Kong‐Macao Joint Laboratory for Neutron Scattering Science and Technology Spallation Neutron Source Science Center Dongguan 523803 China

**Keywords:** anti‐tumor immunity, boron neutron capture therapy, DC vaccine, DNA, radiated tumor cell‐derived extracellular vesicles

## Abstract

Radiated tumor cell‐derived extracellular vesicles (RT‐EVs) encapsulate abundant DNA fragments from irradiated tumor cells, in addition to acting as integrators of multiple tumor antigens. Accumulating evidence indicates these DNA fragments from damaged cells are involved in downstream immune responses, but most of them are degraded in cells before incorporation into derived RT‐EVs, thus the low abundance of DNA fragments limits immune responses of RT‐EVs. Here, this study found that different radiations affected fates of DNA fragments in RT‐EVs. Boron neutron capture therapy (BNCT) induced DNA accumulation in RT‐EVs (BEVs) by causing more DNA breaks and DNA oxidation resisting nuclease degradation. This is attributed to the high‐linear energy transfer (LET) properties of alpha particles from the neutron capture reaction of ^10^B. When being internalized by dendritic cells (DCs), BEVs activated the DNA sensing pathway, resulting in functional enhancements including antigen presentation, migration capacity, and cytokine secretion. After vaccination of the BEVs‐educated DCs (BEV@BMDCs), the effector T cells significantly expanded and infiltrated into tumors, suggesting robust anti‐tumor immune activation. BEV@BMDCs not only effectively inhibited the primary tumor growth and metastasis formation but also elicited long‐term immune memory. In conclusion, a successful DC vaccine is provided as a promising candidate for tumor vaccine.

## Graphical abstract

DNA fragments in radiated tumor cell‐derived extracellular vesicles (RT‐EVs) are mostly degraded, limiting their immune effectiveness. This study finds that boron neutron capture therapy (BNCT) increases DNA accumulation in RT‐EVs (BEVs) by causing DNA breaks and oxidation, resisting nuclease degradation. BEVs improved the function of dendritic cells (DCs) and expands effector T cells, eliciting anti‐tumor immunity and long‐term immune memory.

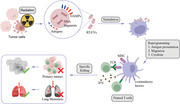

## 1 Introduction

Great progress has been made in the field of tumor vaccines in the past decade, and their therapeutic efficacy relies on the sufficient immune response aroused in vivo.^[^ ^1^, ^2^, ^3^, ^4^, ^5^ ^]^ BNCT, high‐LET radiotherapy based on the neutron capture reaction ^10^B (n, *α*) ^7^Li^,[^ ^6^, ^7^ ^]^ results in the breakage of double‐stranded DNA to destroy tumor cells. In our previous work, BNCT‐treated cancer cells were used as whole‐cell tumor antigens arousing an effective anti‐tumor immune response, achieving good inhibition and even elimination in distal and metastatic tumors.^[^ ^8^ ^]^ Whole tumor cell vaccines (TCVs) as a pool of abundant potential antigens show less susceptibility to tumor escape and provide the possibility of inducing an anti‐tumor immune response.^[^ ^9^, ^10^, ^11^, ^12^ ^]^ Autologous TCVs have been widely studied in clinical trials, but their development is hindered by relatively weak immunogenicity and potential oncogenic risks.^[^ ^13^, ^14^, ^15^, ^16^, ^17^ ^]^

Compared to TCVs, tumor cell‐derived extracellular vesicles (TEVs) avoid the oncogenicity associated with TCVs and retain a wide array of antigens from the entire tumor cell.^[^ ^18^, ^19^, ^20^ ^]^ TEVs hold a diameter of 50–1000 nm and great efficacy due to the unique property to carry proteins and genetic materials including DNA and RNA fragments.^[^ ^21^, ^22^ ^]^ Their smaller size facilitates phagocytosis of antigen‐presenting cells (APCs), enhancing the subsequent antigen presentation process.^[^ ^23^ ^]^ As a common pathogen‐associated molecular pattern (PAMP), DNA is well‐documented to influence the immunogenicity of TEVs.^[^ ^24^, ^25^, ^26^, ^27^, ^28^ ^]^ When double‐stranded DNA appears in the cytoplasm, the cGAS‐STING signaling pathway senses and responds to it, stimulating the expression and secretion of type I interferon (IFN) and downstream interferon‐stimulated genes.^[^ ^29^ ^]^ Radiation can induce immunogenic cell death of tumor cells and produce tumor antigen pools that can initiate anti‐tumor immune responses.^[^ ^30^, ^31^ ^]^ It is worth noting that radiation can generate a large amount of DNA fragments and then possibly encapsulate them in radiated tumor cell‐derived extracellular vesicles (RT‐EVs).^[^ ^32^, ^33^, ^34^ ^]^ However, under normal physiological conditions, DNA fragments are subjected to nuclease degradation within parental cells, thereby diminishing the immunogenicity of RT‐EVs.^[^ ^31^, ^35^ ^]^ For example, X‐ray radiation induced DNA damage but high expression of DNA exonuclease Trex1 degraded cytoplasmic DNA, hindering the recruitment and activation of Batf3‐dependent dendritic cells (DCs) and as well as the following CD8^+^ T cell priming.^[^ ^36^ ^]^ Therefore, the rational design of RT‐EVs to unleash the immune potential of DNA allows for improving their immunogenicity and then the capability of eliciting a sufficient anti‐tumor immune response in vivo.

BNCT as an advanced cell‐selective radiotherapy has shown superior therapeutic efficacy on tumors clinically.^[^ ^37^, ^38^, ^39^ ^]^ Upon capturing neutrons, boron atoms undergo the nuclear reaction ^10^B(n,*α*)^7^Li, where the energy is released through high Linear Energy Transfer (LET) alpha particles within a range of 4.5–9 µm.^[^ ^7^, ^40^ ^]^ Unlike the geometric targeting of therapies such as protons and heavy ions, BNCT achieves cell‐targeted killing through intracellular *α*‐particles, which could induce more complex DNA damage that is more difficult to repair and degrade, and then may produce more DNA fragments in cells.^[^ ^41^, ^42^ ^]^ Whether these abundant DNA fragments could avoid degradation within parental cells and then be encapsulated into RT‐EVs (BEVs) remains to be investigated.

Here, we collected three kinds of RT‐EVs released from non‐ionizing radiation (ultraviolet, UV) and ionizing radiation (X‐ray and BNCT) named UEVs, XEVs, and BEVs, respectively. We found that these three RT‐EVs all harbored a diverse array of tumor antigens and damage‐associated molecular patterns (DAMPs). It was notable that BEVs preserved more DNA fragments due to BNCT producing more DNA breaks and inducing more DNA oxidation that resists nuclease degradation than the others. When incubated with bone marrow‐derived dendritic cells (BMDCs), we achieved a series of RT‐EVs‐educated DC (named UEV@BMDC, XEV@BMDC, and BEV@BMDC). Intriguingly, BEV@BMDC showed the strongest activity to improve antigen presentation, cytokine secretion, and migration capacity, which are the main limitations that impede the clinical efficacy of DC vaccines. Vaccinating BEV@BMDC suppressed the primary tumor growth, prevented the metastatic foci formation, and induced a long‐lasting immune response. The BEV@BMDC provided a path to develop advanced tumor vaccines in the field of cancer immunotherapy.

## 2 Results

### 2.1 Preparation and Characterization of RT‐EVs as a Pool of Tumor Antigens

**Figure 1:**
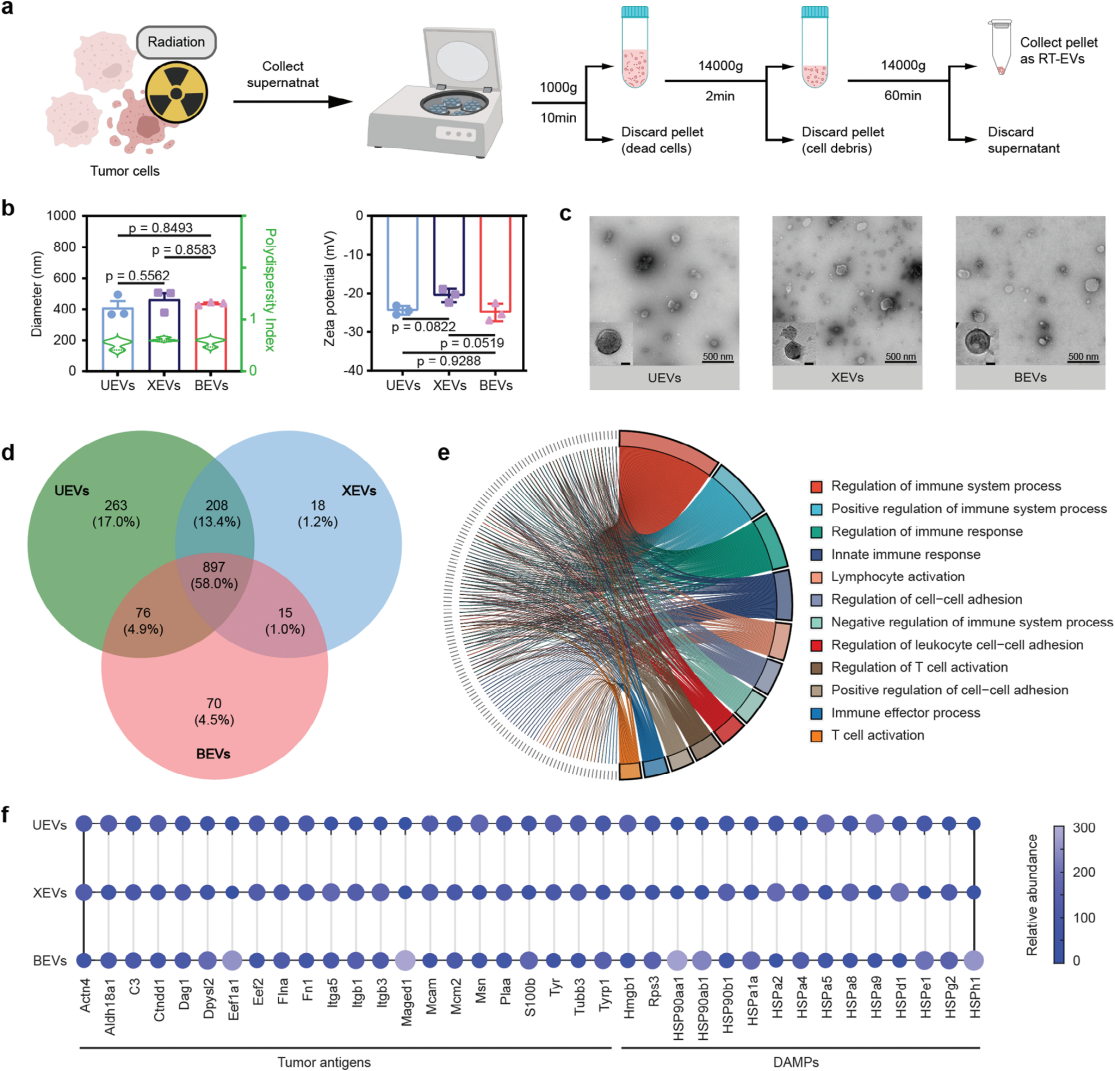
Preparation and characterization of RT‐EVs as a pool of tumor antigens. a) Scheme of RT‐EVs preparation. b) Size and Zeta potential of UEVs, XEVs, and BEVs. c) Representative TEM images of EVs. d) The Venn diagram of proteins overlap among UEVs, XEVs, and BEVs by quantitative proteomic analysis. e) The enriched Gene Ontology (GO) terms in Biological Processes among three kinds of RT‐EVs in the computations in STRING.^[^ ^50^ ^]^ f) Relative abundance of proteins associated with tumor antigens and DAMPs. Data presented as mean ± s.d. (n = 3 biologically independent samples).

In accordance with prior studies, we isolated and gathered three distinct types of RT‐EVs derived from irradiated tumor cells (**Figure** **1**a).^[^ ^34^, ^43^ ^]^ Despite evident differences in particle characteristics among the three radiation types, all these modalities of radiotherapy primarily induce reproductive death in tumor cells through DNA damage.^[^ ^44^, ^45^, ^46^ ^]^ Utilizing the Cell Counting Kit‐8 (CCK‐8), we assessed the cell viability 24 h post‐radiation at various doses and selected a dose that effectively induced tumor cell killing (Figure S1a, Supporting Information). To evaluate the long‐term effects of radiation on cellular proliferative capacity, a canonical clonogenic assay was conducted to calculate the survival fraction, and three radiation doses approximating a survival fraction of 0.1 were selected (Figure S1b‐S1c, Supporting Information).^[^ ^47^ ^]^ Dynamic light scattering revealed comparable sizes of 400 nm and closely matching Zeta potentials of −20 mV for these kinds of RT‐EVs (Figure 1b). Transmission electron microscopy (TEM) illustrated that UEVs, XEVs, and BEVs exhibited a consistently spherical morphology (Figure 1c).

In the role of carriers for endogenous proteins, we conducted a quantitative proteomic analysis to scrutinize the protein composition of the three varieties of RT‐EVs.^[^ ^28^, ^48^, ^49^ ^]^ Figure 1d showed that three kinds of RT‐EVs contained 1547 proteins which could be classified into three categories. The first category comprised proteins unique to each EV type, with counts of 263, 18, and 70 for UEVs, XEVs, and BEVs, respectively. The second category included proteins shared by any two of the EV types, totaling 299 proteins and representing 19.3% of the overall protein content. The third category consisted of proteins common to all three EV types, referred to as overlapped proteins, with a total of 897 proteins, accounting for 58.0% of the total protein content (Figure 1d). Pathway analysis revealed that the overlapped proteins were enriched in pathways related to immune response such as regulation of the immune system process and T cell activation (Figure 1e). In contrast, the other two protein categories were only able to enrich other biological processes (Figure S2, Supporting Information). It is noteworthy that the overlapped proteins carried a diverse array of tumor antigens and damage‐associated molecular patterns (DAMPs) from parental tumor cells without obvious differences, indicating that all RT‐EVs possessed the potential to activate downstream immune signaling pathways (Figure 1f).^[^ ^26^ ^]^

### 2.2 Accumulation of DNA Fragments in BEVs

**Figure 2:**
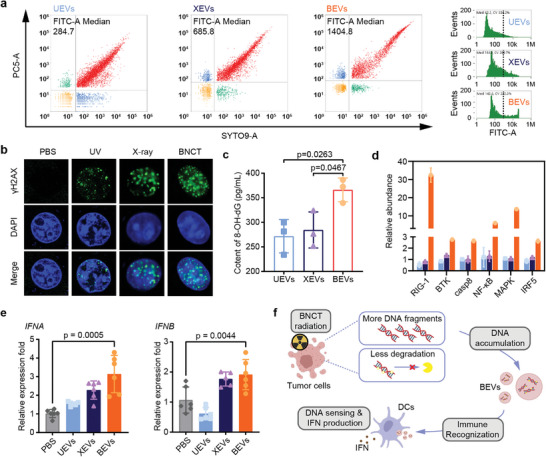
Boosted immune recognition from abundant DNA fragments within BEVs. a) nano‐flow cytometry of three kinds of EVs. SYTO9 represents DNA fragments and PC5 labels lipid membrane of EVs. b) CLSM images of DNA damage (*γ*‐H2AX) within irradiated tumor cells. c) content of 8‐OH‐dG measured by ELISA kit. d) proteins related to nucleic acids sensing pathway in EVs‐educated BMDCs. e) BMDCs were incubated with EVs. IFN*α* and IFN*β* mRNAs were assessed by real‐time PCR 24 h later. f) scheme of accumulation of abundant DNA fragments within BEVs.

DNA is well‐documented to stimulate immune response and influences the immunogenicity of EVs.^[^ ^24^, ^25^, ^26^, ^27^ ^]^ Different radiations result in varied fates of DNA. To investigate whether there are differences in DNA fragments in these three RT‐EVs, we detected DNA fragments with SYTO9 staining in EVs using a nanoflow cytometry. It was observed that BEVs contained a larger quantity of DNA fragments than UEVs and XEVs. The abundance of DNA fragments increased by ≈4.93 and 2.05 folds, respectively (**Figure** **2**a). To reveal the mechanism, we first determined the DNA breaks in cells after different radiations. As shown in Figure 2b, a substantial amount of DNA damage is generated in the cells post‐radiation, especially in BNCT‐treated cells, which may contribute to its high‐LET nature. In normal physiological conditions, these DNA fragments are degraded by cellular nucleases, thereby preventing the occurrence of immune sensing of DNA.^[^ ^36^ ^]^ It has been reported that oxidation‐induced structural alterations in DNA molecules provided resistance against cytosolic exonuclease TREX1, leading to a delayed degradation of oxidative DNA fragments.^[^ ^36^, ^51^ ^]^ Then we quantified the content of the oxidated DNA in BEVs with a 8‐OH‐dG detection kit because that the guanine base in nucleic acids is the most vulnerable to producing 8‐oxoguanine.^[^ ^52^ ^]^ We observed a significantly higher content of oxidized DNA fragments in BEVs compared to UEVs and XEVs (Figure 2c), suggesting that DNA resists to be degraded after BNCT. Therefore, more DNA fragments production and less degradation after BNCT collectively promote DNA accumulation and being packaged within BEVs. These DNA fragments act as typical pathogen associated molecule pattern (PAMP) and have the capacity to activate the immune system.^[^ ^29^, ^35^, ^53^ ^]^

Followed by internalization of BEVs by BMDCs, it was observed that functional proteins associated with DNA sensing pathway substantially upregulated in BEV@BMDC in comparison to UEV@BMDC and XEV@BMDC (Figure 2d). Since DNA has been reported to induce the release of pro‐inflammatory cytokines from immune cells, we examined interferon levels in BMDCs.^[^ ^53^, ^54^ ^]^ As a result, BEVs markedly induced the expression of interferon *α* (IFN*α*) and interferon *β* (IFN*β*) in BMDCs (Figure 2e). In summary, BNCT achieved accumulation of DNA fragments within BEVs elevated the immunogenicity of BEVs, which laid the foundation for developing BEVs as an excellent tumor vaccine (Figure 2f).

### 2.3 Functional Enhancement of DCs Caused by BEVs

**Figure 3:**
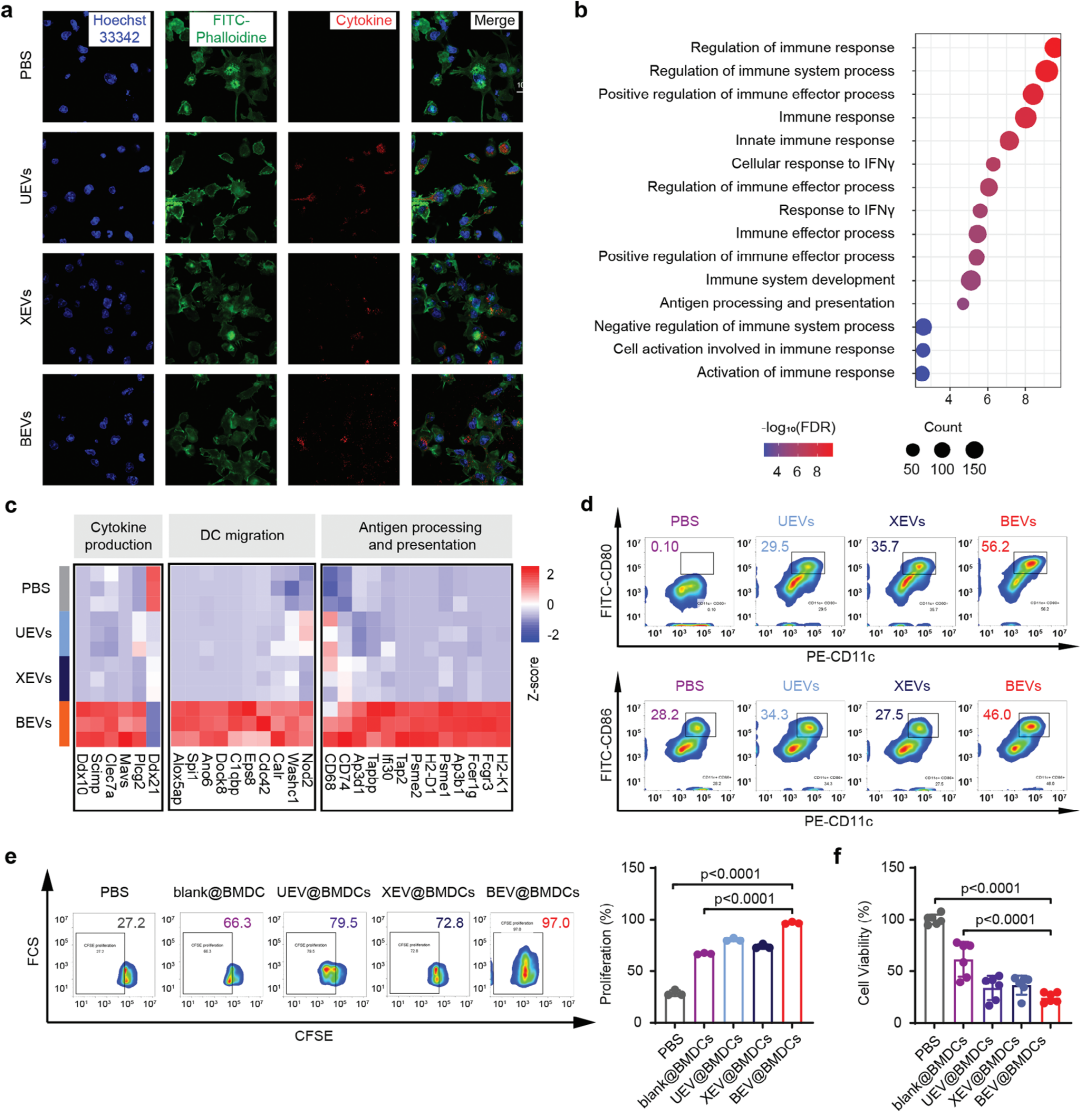
The mechanism by which BEV boosts DC vaccine efficacy. a) CLSM images of engulfment of EVs. b) Graphical representation of the enrichment of gene ontology terms for immune response in BEV@BMDC group. c) Heat map of proteins in antigen processing and presentation, DC migration, and cytokine production. d) DC maturation measured by FITC‐CD80 and FITC‐CD86 by flow cytometry. e) T‐cell proliferation was examined by carboxyfluorescein diacetate succinimidyl ester (CFSE) assay followed by co‐culture with EVs pulsed BMDCs. f) Tumor‐specific killing percentage detected by Cell Counting Kit‐8 (CCK‐8) assays.

To verify whether the accumulated DNA in BEVs promotes the immune response, we employed an ex vivo incubation method to observe and evaluate the uptake of these RT‐EVs by BMDCs and followed the antigen presentation process.^[^ ^18^ ^]^ Fluorescent images illustrated that various EVs were effectively endocytosed, and flow cytometry showed no significant difference in the quantity of internalization (**Figure** **3**a; Figure S3, Supporting Information). Following a 24‐h incubation, the EVs‐pulsed BMDCs were collected for quantitative proteomics analysis. BEV@BMDC exhibited an upregulation of 1867 proteins out of all detected 2212 proteins, in contrast to PBS‐treated BMDCs, while only 198 and 150 proteins were upregulated in UEV@BMDC and XEV@BMDC, respectively (Figure S4, Supporting Information). These upregulated proteins were subjected to Gene Ontology (GO) analysis for biological processes. Compared with UEV@BMDC and XEV@BMDC, only the upregulated proteins of BEV@BMDC were enriched in multiple immune response‐related pathways (Figure 3b; Figure S5, Supporting Information). Notably, BEVs upregulated numerous proteins in BMDCs associated with antigen processing and presentation, migration capacity, and cytokine production (Figure 3c). These aspects have been reported to contribute to the unsatisfactory clinical outcomes of DC vaccines.^[^ ^55^, ^56^, ^57^, ^58^ ^]^ Meanwhile, BEVs elevated the expression of MHC class I, CD80, and CD86, suggesting that BEVs prompted the maturation of DCs (Figure 3d; Figure S6, Supporting Information). All the above results indicate that BEVs with greater DNA accumulation have higher immunogenicity and can effectively enhanced function of DC vaccines.

To investigate the immune activation efficacy of RT‐EVs‐educated DCs, we isolated the spleens of mice immunized with these DC vaccines to evaluate the proliferation ability and specific killing ability of T cells ex vivo. The percentage of proliferated T cells in spleens stimulated by the BEV@BMDC was 3.17 times and 53.5 times higher than that of UEV@BMDC and XEV@BMDC, respectively (Figure 3e,f). To preliminarily validate the immunological efficacy of the DC vaccine, mice were inoculated with EVs‐treated BMDCs. Following vaccination, their splenocytes were isolated and collected to assess proliferative and cytotoxic capabilities.^[^ ^59^, ^60^ ^]^ Accordingly, 75.27% of B16‐F10 tumor cells were killed by splenocytes from BEV@BMDC immunized mice, exhibiting greater cytotoxic effect than 66.04% of UEV@BMDC and 63.37% of XEV@BMDC (Figure 3f). It implied that BEV@BMDCs could stimulate T cells and trigger adaptive immunity in vivo, thus becoming a promising tumor vaccine.

### 2.4 Primary Tumor Growth Inhibition Induced by BEV@BMDC Vaccine

**Figure 4:**
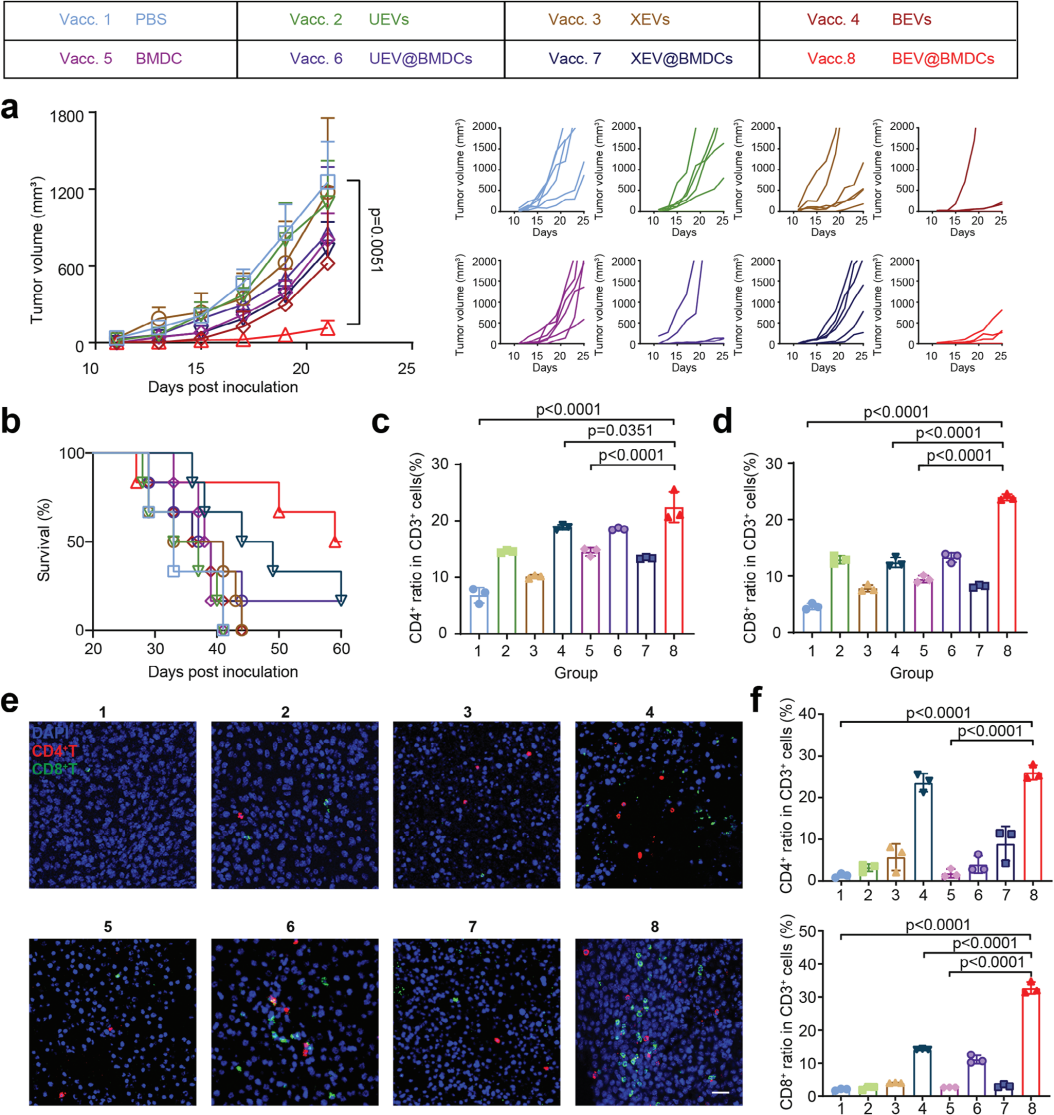
Therapeutic effect with vaccines in solid tumor models. a) Tumor growth curves B16‐F10 cells subcutaneous transplant model in corresponding treatment groups. The tumor volume was monitored every 2 days. Data were shown as means ± s.d. (n = 6) from six independent animals. b) Kaplan–Meier survival curves of mice in different treatment groups (n = 6) from six independent animals. c) CD4^+^ T cell populations in spleen from different groups were detected using flow cytometry d) CD8^+^ T cell populations in spleen from different groups detected using flow cytometry. e) CD4^+^ T cells and CD8^+^ T cells levels were detected by immunostaining in tumor paraffin section slides. Representative images from random fields of view from one of six biologically independent animals. Scale bar, 20 µm. f) CD4^+^ T cell and CD8^+^ T cell populations in tumor sites from different groups were detected using flow cytometry.

We further accessed the therapeutic effect of vaccine candidates in mice subcutaneously inoculated with B16‐F10 (**Figure** **4** List). UEVs, XEVs, and BEVs were administrated at an equal protein concentration detected by BCA assay, respectively. UEV@BMDCs, XEV@BMDCs, and BEV@BMDCs were given at the same counts of BMDCs (1 × 10^6^ cells) pulsed by the same protein concentration EVs. All vaccine candidates were subcutaneously injected three times in each group followed by inoculation of B16‐F10 tumor cells on Day 0. Comparing with PBS treatment, BEV@BMDCs exhibited the greatest tumor volume reduction (89.2%) in all candidates (Figure 4a), indicating BEV@BMDC possessed the most robust anti‐tumor activity. Meanwhile, Kaplan–Meier survival analysis showed that the mice from Vacc.1 to Vacc.5 all died before Day 44. However, 50% of mice in BEV@BMDCs were still alive until Day 60, better than 16.7% in both UEV@BMDCs and XEV@BMDCs (Figure 4b). In addition, body weight change and H&E staining of major organs showed that all candidates did not cause any apparent systemic toxicity (Figures S7 and S8, Supporting Information).

We speculated that tumor inhibition correlated with the activated immune system by DC vaccines. To verify this hypothesis, suspend single cells from the spleen tissues of immunized mice were collected to test the T cell subtype population. Comparing with PBS group, the proportion of CD4^+^ and CD8^+^ T cells slightly added in these treatments except BEV@BMDCs. While BEV@BMDCs treatment increased the proportion of CD4^+^ and CD8^+^ T cells 3.3‐fold and 5.2‐fold, respectively (Figure 4c,d; Figure S9, Supporting Information). Capacity of T cell infiltration in tumor tissue is positive correlation with their activity of anti‐tumor activity. Intriguingly, BEV@BMDCs induced CD4^+^ T cells and CD8^+^ T cells to infiltrate in deep layer of tumor tissue (Figure 4e). Via flow cytometry, the proportions of CD4^+^ T cells and CD8^+^ T cells in the BEV@BMDCs group dramatically raised 21.7‐fold and 16.3‐fold in tumor tissues compared with the control group, respectively (Figure 4f; Figure S10, Supporting Information). Meanwhile, the ratio of regulatory T cells, a major kind of immunosuppressive cells decreased notably in the BEV@BMDCs group (Figure S11, Supporting Information). Therefore, BEV@BMDCs generated the strongest T cell response and greatly activated the effective adaptive immune, which eventually inhibited primary tumor growth.

### 2.5 Metastasis Prevention Caused by BEV@BMDCs Vaccine

**Figure 5:**
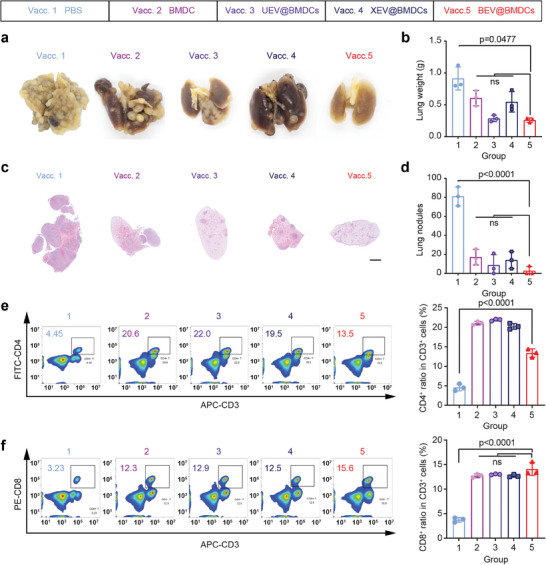
Therapeutic effect with vaccines in metastasis models. a) Representative ex vivo images of the lung with metastatic foci from different groups. b) Lung mass measured after tumor challenge. c) H&E staining of the vaccinated mouse lungs collected after tumor inoculation. The nuclei‐rich regions represent tumor metastases. d) The statistic result of the number of lung nodules. Data were shown as means ± s.d. (n = 3) from three independent animals. e) CD4^+^ cell populations in spleens from different groups were detected using flow cytometry. f) CD8^+^ T cell populations in spleens from different groups were detected using flow cytometry. Statistical significance was obtained by one‐way ANOVA.

Encouraged by outstanding inhibition for solid tumor growth, we further employed BEV@BMDCs in B16‐F10 tumor metastasis models. Similar to before, vaccine candidates were subcutaneously injected three times. The representative lung pictures showed that BEV@BMDCs could obviously retard metastasis (**Figure** **5**a; Figure S12a, Supporting Information). With the formation of metastatic foci, the lung weight in PBS treatment added to 0.91 ± 0.18 g, and plenty of metastatic nodules were formed while BEV@BMDCs obviously lessened the lung weight to 0.26 ± 0.04 g and the nodules reduced apparently (Figure 5b–d; Figure S12b–d, Supporting Information). Besides, BEV@BMDC‐treated mice maintained normal body weight and survived much longer than the other vaccine candidates (Figure S13, Supporting Information).

We further investigated the immune response in vivo by monitoring the T cell population. The results demonstrated that the percentage of CD4^+^ T cells in the spleen expanded from 4.56 ± 0.90% in the PBS group to 13.40 ± 1.05% in the BEV@BMDCs group (Figure 5e; Figure S14a, Supporting Information). Likewise, the percentage of CD8^+^ T cells after BEV@BMDCs treatment multiplied 3.8‐fold from the PBS group to 14.10 ± 1.30% (Figure 5f; Figure S14b, Supporting Information). The prominent expansion was attributed to the powerful immune responses of the BEV@BMDCs. Additionally, H&E staining analysis confirmed that all vaccines exerted no obviously harmful influence on animals (Figure S15, Supporting Information). In conclusion, the BEV@BMDCs presents the potential value to generate an outstanding antineoplastic immune response to restrain tumor metastasis in monotherapy.

### 2.6 BEV@BMDCs Vaccine Strengthens the Long‐Term Anti‐Tumor Immune Response

**Figure 6:**
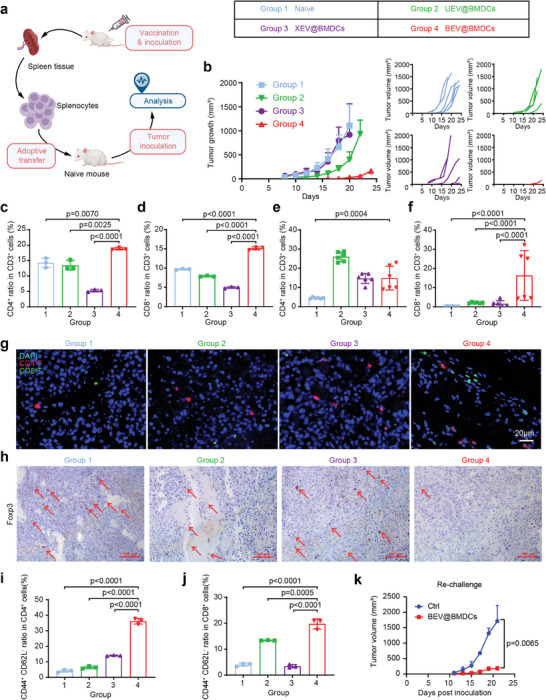
The immunity adoptive experiment. a) Experimental outline of the adoptively transferred model. b) The tumor growth curves B16‐F10 cells adoptive models in corresponding treatment groups. The tumor volume was monitored every 2 days. Data were shown as means ± s.d. (n = 6) from six independent animals. c) CD4^+^ T cell populations in spleens from different groups detected using flow cytometry. d) CD8^+^ T cell populations in spleens from different groups detected using flow cytometry. e) CD4^+^ T cell populations in tumor tissues from different groups were detected using flow cytometry. f) CD8^+^ T cell populations in tumor tissues from different groups were detected using flow cytometry. g) CD4^+^ T cell and CD8^+^ T cell levels were detected by immunostaining in tumors paraffin section slides. Representative images from random fields of view from one of six biologically independent animals. Scale bar, 20 µm. h) T_reg_ cells (Foxp3 positive) levels were detected by immunohistochemical assay in tumors paraffin section slides. Representative images from random fields of view from one of six biologically independent animals. Scale bar, 100 µm. i) Proportions of CD44^+^CD62L^−^ cells in CD4^+^ T cells of tumor tissues. j) Proportions of CD44^+^CD62L^−^ cells in CD8^+^ T cells of tumor tissues. k) Tumor growth curves of re‐challenge models (B16‐F10) of Ctrl group and BEV@BMDCs group (n = 3). Statistical significance was obtained by one‐way ANOVA.

To evaluate the long‐term anti‐tumor immune response, we used an adoptive transfer model of mice (**Figure** **6**a). Splenocytes (4 × 10^6^) from unimmunized mice were considered as a naïve control and were then intravenously injected into naïve mice named Group 1. Similarly, splenocytes extracted from survived mice treated by UEV@BMDCs, XEV@BMDCs, and BEV@BMDCs and were transferred to naïve mice (named Group 2, Group 3, and Group 4 respectively) (Figure 6, List). After 2 days, each of the transferred mice was implanted with B16‐F10 tumor cells (1 × 10^5^) followed by monitoring and analysis. In comparison, Group 4 inhibited 90.3% of tumor growth while Group 2 and Group 3 exhibited effective scarcely to inhibit on tumor progression (Figure 6b). In the spleen tissues of adoptive mice, the proportion of CD4^+^ T cells in Group 4 was elevated from 14.27 ± 1.55% of Group 1 to 18.87 ± 0.37%, and the ratio of CD8^+^ T cells increased 1.6‐fold from 9.70 ± 0.17% of Group 1 to 15.13±0.38% (Figure 6c,d; Figure S16, Supporting Information). The results represented the efficient activation of systemic immune responses in Group 4. Moreover, histological examinations of the main organs with H&E staining exhibited that the adoptive transfer had no adverse impression on each animal (Figure S17, Supporting Information).

Compared with Group 1, Group 2, and Group 3 have a slight improvement in the infiltration of CD4^+^ T cells and CD8^+^ T cells in tumors. Whereas, Group 4 showed an obvious infiltration of T lymphocytes (Figure 6g). The flow cytometry quantified that the percentage of CD4^+^ T cells in tumor tissue of Group 4 mice was 3.3‐fold of control group. The proportion of CD8^+^ T cells in Group 1 was merely 0.26 ± 0.10% while Group 4 increased dramatically 62.4‐fold after transferring (Figure 6e,f; Figure S18, Supporting Information). To further evaluate the specific cytotoxic capability of CD8^+^ T cells induced by BEV@BMDCs immunization in vivo, we inoculated the MB49 subcutaneous tumor models in adoptive models. The results indicated that, compared to the control group, BEV@BMDCs did not effectively inhibit the growth of MB49 tumors (Figure S19, Supporting Information). Additionally, Group 4 displayed lowest level of immunosuppressive response through reduced Treg cells (Foxp 3 positive cells) compared with other groups (Figure 6h).

Effector memory T cells (T_em_), involved in long‐term anti‐tumor immune response, primarily activate CD8^+^ T cells and possess the capacity to augment immunoreaction after the re‐introduction of relevant antigens into the body. We further investigated the percentage of T_em_ (CD44^+^CD62L^−^) in tumor tissues via flow cytometry. CD4^+^ T_em_ and CD8^+^ T_em_ in Group 4 exhibited an 8.8‐fold and a 4.9‐fold increase compared with the control group, respectively (Figure 6i,j; Figure S20, Supporting Information). To evaluate long‐term immune memory and persistence of therapeutic effects of DC vaccines, survived mice of BEV@BMDCs were involved secondary exposure of B16‐F10 tumor cells inoculation as re‐challenge models.^[^ ^2^, ^61^ ^]^ The results showed that tumor growth was obviously suppressed in the re‐challenge models of BEV@BMDCs (Figure 6k; Figure S21, Supporting Information). In summary, the data in this study revealed that the BEV@BMDCs vaccine not only induced a robust anti‐cancer immune response in the solid tumor model but also generated long‐term memory immunity.

## 3 Discussion

We selected boron neutron capture therapy (BNCT) different from other radiation types to treat tumor cells to gain radiated cells‐derived extracellular vesicles (TEVs). It is noteworthy that BNCT generated more DNA fragments and induced oxidation of DNA in treated cells, and then inherits these DNA fragments to BEVs. After internalization by BMDCs, these DNA molecules acted as adjuvants to upregulate DNA sensing proteins and induced pro‐inflammatory factors production. BMDCs pulsed by BEVs, as a tumor vaccine candidate (named BEV@BMDCs), elicited strong antitumor immunity in vivo. After immunized mice with BEV@BMDCs, it not only induced systemic immunity to effectively inhibit the growth of primary tumors and the formation of metastases, but also produced a long‐term immune memory effect. Harnessing the intrinsic properties of high‐LET radiation of BNCT, a series of highly efficient tumor vaccine methods could be developed, which broadens the potential and scope of BNCT in clinical.

## 4 Experimental Section

### Materials

Polyvinylpyrrolidone (PVP) was obtained from Aladdin. Boric acid (H_3_BO_3_) and urea was purchased from Macklin reagent Co., Ltd. Imiquimod (R837) was purchased from MedChemExpress (USA). FITC‐anti‐mouse CD4 (#100509), APC‐anti‐mouse CD44 (#103012), PerCP/Cyanine5.5‐anti‐mouse CD62L (#104431), APC‐anti‐mouse CD3ε (#100312) and PE‐anti‐mouse CD8a (#100708) were purchased from Dakewe Biotech Co., Ltd (Beijing, China). PE‐anti‐CD80 Antibody (#E‐AB‐F0992J) and FITC‐anti‐CD86 (#105005) were obtained from Elabscience. 20 mm glass‐bottom dishes were bought from NEST Biotechnology Co., Ltd. Hoechst33342 was purchased from Lablead Inc. (Beijing, China). Annexin V‐FITC and PI were Research Science Biotechnology Co., Ltd. (Shanghai, China).

### Animals and Cells

C57BL/6 (female and male, 18–22 g) were provided by the Vital River Laboratory Animal Technology Co. Ltd (Beijing, China). All animal procedures were approved by the Administrative Panel on Laboratory Animal Care at the Institute of High Energy Physics, Chinese Academy of Medical Sciences (IHEPLLSC202018). Murine melanoma cells (B16‐F10) and DC2.4 cells were obtained from the National Center for Nanoscience and Technology as a gift. B16‐F10 cells were cultured in DMEM high glucose medium supplemented with 10% fetal bovine serum (FBS, TransSerum FQ Fetal Bovine Serum, China). All cells were cultured in a humidified environment of 37 °C and 5% CO_2_.

### Induction and Culture of Mouse BMDCs

Bone marrow‐derived dendritic cells (BMDCs) were generated by isolating bone marrow cells from male C57BL/6 mice according to the previous report.^[^ ^18^, ^62^ ^]^ Briefly, tibia and femurs were dissected from mice. The bone marrow cells were collected and treated by red blood cell lysis buffer. The obtained cells were cultured in a medium containing 20 ng·mL^−1^ GM‐CSF and 12.5 ngmL^−1^ IL‐4 for 5–6 days. The culture medium was changed every 2 days and cytokines were supplemented. Cells were cultured in a humidified environment of 37 °C and 5% CO_2_.

### Clonogenic Assay

B16‐F10 cells were cultured in DMEM medium supplemented with 10% fetal bovine serum, 100 units per mL penicillin, and 100 µg mL^−1^ streptomycin at 37 °C in a humidified atmosphere containing 5% CO_2_. For radiation experiments, cells were seeded 6‐well plates at a density of 1000 cells per well and maintained under standard culture conditions for 14 days, allowing colonies to form. At the end of the incubation period, colonies were fixed with 4% paraformaldehyde and stained with 0.5% crystal violet for 15 min. The plates were washed and air‐dried. Colonies consisting of more than 50 cells were counted using ImageJ software.

### Preparation of RT‐EVs

First, all the tumor cells were cultured into 10 or 15‐cm dishes and maintained in medium containing 10% FBS, 100 U mL^−1^ penicillin and 100 µg mL^−1^ streptomycin at 37 °C and in a 5% CO_2_ incubator. To collect EVs, the cells were irradiated by UV (UVB, 300 J m^−2^) for 1 h.^[^ ^18^ ^]^ Discard the irradiated culture medium and supply 10 mL fresh culture medium with 10% FBS per 10 cm dish or 25 mL fresh culture medium with 10% FBS per 15 cm dish. After 72 h‐incubation, the culture medium was collected for EVs isolation.^[^ ^18^, ^23^ ^]^ Briefly, supernatants were centrifuged at 1000 g for 10 min at 4 °C to remove whole cells and then centrifuged for 2 min at 14 000 g at 4 °C to remove debris. The supernatant was collected and further centrifuged at 14 000 g for 60 min 4 °C to pellet RT‐EVs. The pellets were washed three times, centrifuged at 14 000 g for 60 min 4 °C to pellet, and resuspended in PBS. Similar to the steps above, XEVs were collected except the cells were plated into 10‐cm cell culture dishes were irradiated with a single dose of 20 Gy X‐rays (160‐kV, 25 mA) as described previously.^[^ ^34^ ^]^ As for BEVs, the cells were treated with 100 µg mL^−1^ boron nitride nanoparticles. At 24 h after incubation, the cells were irradiated with neutrons (2.5 kW, 2.57 × 10^8^ cm^−2^·s^−1^ for 3 h).^[^ ^63^ ^]^ All the radiated medium were changed to fresh medium with 10% FBS, 100 U mL^−1^ penicillin and 100 µg mL^−1^ streptomycin. Other steps were exactly same with UEVs and XEVs.

### Characterization and Quantification of EVs

The protein concentration of three kinds of EVs were measured by BCA protein assay kit (Beyotime Biotechnology) in accordance with the manufacturer's protocol. Briefly, EVs were treated by lysing buffer (RIPA and PMSF) at 4 °C overnight. Supernatant contained total protein was detected by microplate reader at 562 nm. EVs in suspension deposited on copper meshes and then stained with uranyl acetate for 15 s followed by deionized water wash three times. After dehydration at room temperature, the morphology of EVs was observed by TEM. The hydrodynamic size and Zeta Potential of particles were recorded on a dynamic light scattering (Zetasizer, Malvern Instruments, UK) at 25 °C.

### LC‐MS/MS Proteomics Analysis

The tryptic peptides were analyzed using an UltiMate 3000 nano‐LC system coupled with an Orbitrap Fusion Lumos Mass Spectrometer (Thermo Fisher Scientific, USA). EVs and BMDCs were ultrasonic disrupted in RIPA buffer under ice bath. Protein content in cell lysis buffers were quantified by BCA kit, and 100 µg protein for each sample was collected for proteomics analysis. Samples were reduced by dithiothreitol (DTT) and alkylated by iodoacetamide (IAA), and then were incubated with trypsin to digested into peptides overnight at 37 °C. Peptide solution was desalted on C18 column. Finally, samples were analyzed by LC‐MS/MS (Thermo Orbitrap Fusion Lumos). The original mass spectrometry data collected by the Xcalibur analysis system were searched and quantified by Proteome Discoverer (version 2.4, Thermo Fisher Scientific), and the peptide matching and protein identification were carried out by Sequest HT algorithm against the bovine SwissProt reviewed database (June 2022, 6017 entries). The following search parameters were used: peptide mass tolerance, 20 ppm; fragment mass tolerance, 0.1 Da; digest enzyme: trypsin/P; the number of missed cleavages, up to three; carbamidomethyl (C) as the fixed modification; methionine oxidation (M) and acetyl (protein N‐term) as the variable modifications. Proteins were identified based on one or more unique tryptic peptides with 95% confidence (*p* < 0.05). The false discovery rate (FDR) of the peptide and protein identifications was set as < 1%, as determined by automatically searching a decoy database. Label free‐quantification (LFQ) was used to calculate the protein abundance ratio with an alignment time window of 5 min and ratio calculation of pairwise ratio based using the Proteome Discoverer software.

### Cell Uptake

BMDCs were treated with DiR labeled UEVs, XEVs and BEVs at 73 µg mL^−1^ for 24 h. The cells were then rinsed with PBS three times. Hoechst33342 was used to stain the nucleus and FITC‐phalloidine was indicated the cytoplasm. Then images were captured using a confocal laser‐scanning microscope. All image analyses were performed using Image J Software and Nikon software.

### In Vitro BMDC Maturation

BMDCs were treated with UEVs, XEVs and BEVs at 73 µg mL^−1^ for 24 h. After incubation,APC‐H2, PE‐CD80 (Biolegend, cat. No 104708) and FITC‐CD86 (Biolegend, cat. No 105012), and then detected by flow cytometry (Accuri C6, BD, USA) and analyzed by CFlow plus software and Flowjo (BD Biosciences) software.

### Immunization and Tumor Challenge Setting

C57BL/6 were immunized three times in the lower left flank with PBS, UMPs, XMPs, BMPs, blank@BMDCs, UMP@BMDCs, XMP@BMDCs and BMP@BMDCs by intramuscular injection on day −28, day −21, and day −7. MPs were injected at 7.3 µg each mouse while BMDCs were injected at 1 × 10^6^ cells each mouse. Then immunized mice were inoculated B16‐F10 tumor cells (1 × 10^6^ cells per mouse) in the right flank. Tumors and body weight were monitored by calipers every two or three days. And tumor volume was calculated according to the formula: volume = (length × width^2^)/2, in which length and width were measured in millimeters. Mice were sacrificed when the tumor size reached 2500 mm^3^ or when mice became moribund with severe weight loss. In the metastasis model, C57BL/6 mice were i.v injected with 1 × 10^5^ B16‐F10 cells after immunization. Survival and body weight of these mice were monitored every two days. Lungs were collected, fixed in 4% paraformaldehyde and sectioned for H&E staining. The metastatic tumor nodules in the lungs were counted and performed statistical analysis.

### Immunomonitoring of Tumor‐Bearing Mice

To analyze changes of immune cells in tumor immune environment (TME) and lymphoid organ of mice, spleen and tumor tissues were collected respectively from distinct mice. Red blood cells were lysed and single cells from tissues were separated for APC‐CD3ε, FITC‐CD4 and PE‐CD8 staining. Then the cell samples were analyzed using a flow cytometer. The data was analyzed by CFlow plus software and Flowjo (BD Biosciences) software.

### T‐Cell Proliferation Assay

Splenocytes were purified from B16 tumor–bearing mice and then labeled with 5 mmol L^−1^ CFSE (Sigma–Aldrich). BMDCs were incubated with accordingly MPs for 24 h. CFSE‐labeled splenocytes were incubated MP‐loaded BMDCs, or empty BMDCs for 72 h before flow cytometric analysis.

### Tumor‐Specific CTL Killing Assay

A tumor‐specific CTL killing assay was performed as described previously.^[^ ^18^ ^]^ Briefly, splenocytes were harvested from mice pre‐immunized after tumor implantation, and single‐cell suspensions were prepared to co‐culture with tumor cells (splenocytes: tumor cells = 1:20). 72 h later, the cells were harvested and used as CTL effector cells in a Cell Counting Kit assay. The percentage of specific killing was defined as (experimental value − blank control value) ÷ (PBS‐treated value − blank control value) × 100%

### Adoptive Transfer

To demonstrate immune memory, mice that had survived the immune study were used in this experiment. After killing the survivors and collecting their spleen tissues, red blood cells were lysed and single cells were obtained to intravenous injected into naïve mice (4 × 10^6^ cells per mouse). And three unimmunized animals were injected with spleen cells from untreated animals as control group. Two days later, each group was subcutaneously injected with 1 × 10^5^ B16‐F10 cells for challenge. Tumor volume and body weight were monitored every other day. The size of tumors and weight of mice were recorded every each other. Tumor volume was calculated as width^2^ × length/2. Mice were sacrificed when the tumor size reached 2500 mm^3^ or when mice became moribund with severe weight loss. Single cells were obtained from spleen tissues and tumor tissues of adoptive mice to analyze CD4^+^ T cells (helper T cells), CD8^+^ T cells (cytotoxic T‐lymphocyte cells), CD4^+^ central memory T cells (CD4^+^ T_cm_: CD4^+^CD44^+^CD62L^+^), CD8^+^ central memory T cells (CD8^+^T_cm_: CD8^+^CD44^+^CD62L^+^), CD4^+^ effect memory T cells (CD4^+^ T_em_: CD4^+^CD44^+^CD62L^+^) and CD8^+^ effect memory T cells (CD8^+^ T_em_: CD8^+^CD44^+^CD62L^−^) by flow cytometry analysis (Accuri C6, BD, USA).

### H&E Staining, Immunohistochemical and Immunofluorescence Staining

The major organs and tumor tissues of mice in different treatment groups were collected, fixed with 4% paraformaldehyde at room temperature, and cut into 5‐µm sections for analysis. Tissue sections were stained with Hematoxylin and eosin (H&E). Images were captured by a bright field microscope. For immunohistochemical staining, the tumor slices were incubated with foxp3 antibodies, followed by staining with the horseradish‐peroxidase‐conjugated IgG antibody according to the manufacturers protocols. Immunofluorescence images of CD8^+^ T cells (labeling with Cy5.5‐conjugated CD8 antibody, red) and CD4^+^ T cells (labeling with FITC‐conjugated CD4 antibody, green) in tumor tissues of B16‐bearing mice were acquired using Pannoramic Scanner (3DHIS‐TECH, Hungary) and analyzed using CaseViewer (3DHISTECH, Hungary) and StrataQuest tissue analysis software (TissueGnostics, Asia Pacific limited, China).

### Statistical Analysis

All the results were shown as mean ± s.d. Data were carried out with the IBM SPSS statistics (Chicago, USA). Differences between groups were analyzed using analysis of variance (ANOVA). Statistical significance thresholds were set at ^*^ *p* < 0.05, ^**^ *p* < 0.01, ^***^ *p* < 0.001, ^****^p<0.0001.

## Conflict of Interest

The authors declare no conflict of interest.

## Supporting information

Supporting Information

## Data Availability

Research data are not shared.
