# Supplementary material for: Boron Neutron Capture Therapy‐Derived Extracellular Vesicles via DNA Accumulation Boost Antitumor Dendritic Cell Vaccine Efficacy
Source: Adv Sci (Weinh). 2024 Jul 17;11(35):2405158. doi: 10.1002/advs.202405158 (PMC11425286; doi:10.1002/advs.202405158)
Supplement: Supplementary file 1 — Supporting Information [file ADVS-11-2405158-s001.docx]

**Boron Neutron Capture Therapy-Derived Extracellular Vesicles via DNA Accumulation Boost Antitumor Dendritic Cell Vaccine Efficacy**

Linwen Lv^1,4^, Junzhe Zhang^2^, Yujiao Wang^1^, Haojun Liang^1^, Qiuyang Liu^1^, Fan Hu^1^, Hao Li^1^, Wenxi Su^1^, Junhui Zhang^1^, Ranran Chen^1^, Ziteng Chen^1^, Zhijie Wang^1^, Jiacheng Li^1^, Ruyu Yan^1^, Mingxin Yang^1^, Ya-nan Chang^1^, Juan Li^1^, Tianjiao Liang^3*^, Gengmei Xing^1*^, Kui Chen^1*^

^1^ CAS Key Lab for Biomedical Effects of Nanomaterials and Nanosafety, Institute of High Energy Physics, Chinese Academy of Sciences, 19B YuquanLu, Shijingshan District Beijing, 100049, China

^2^ State Key Laboratory for Quality Ensurance and Sustainable Use of Dao-di Herbs, Artemisinin Research Center, and Institute of Chinese Materia Medica, China Academy of Chinese Medical Sciences, Beijing 100700, China

^3^ Guangdong-Hong Kong-Macao Joint Laboratory for Neutron Scattering Science and Technology, Spallation Neutron Source Science Center, Dongguan 523803, China

^4^ University of Chinese Academy of Sciences, Beijing 100049, China

**Supplementary Information**


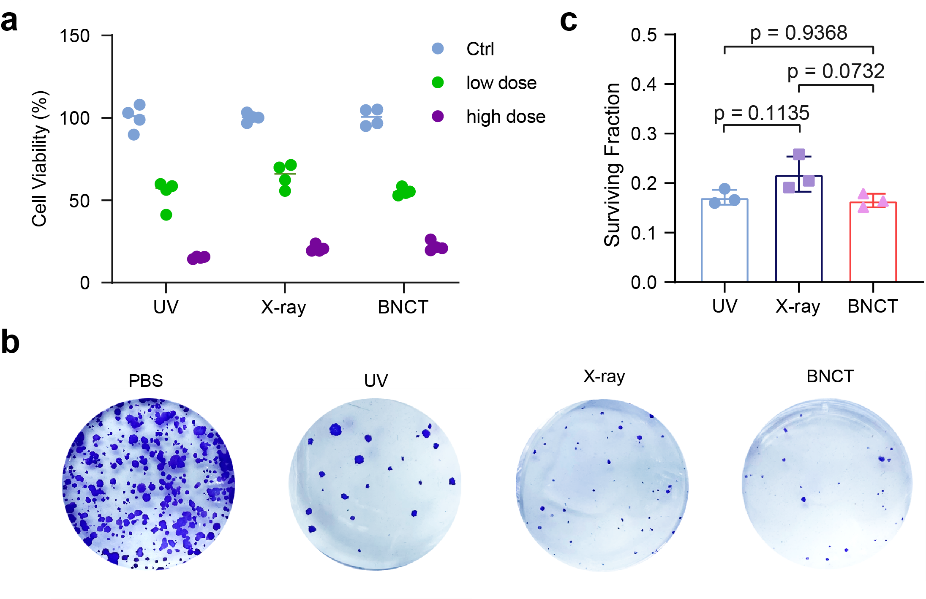


**Supplementary Figure 1** Cell viability **(a)**, colony formation images **(b)** and surviving fraction **(c)** of tumor cells receiving ultraviolet, X-ray and neutron irradiation respectively.


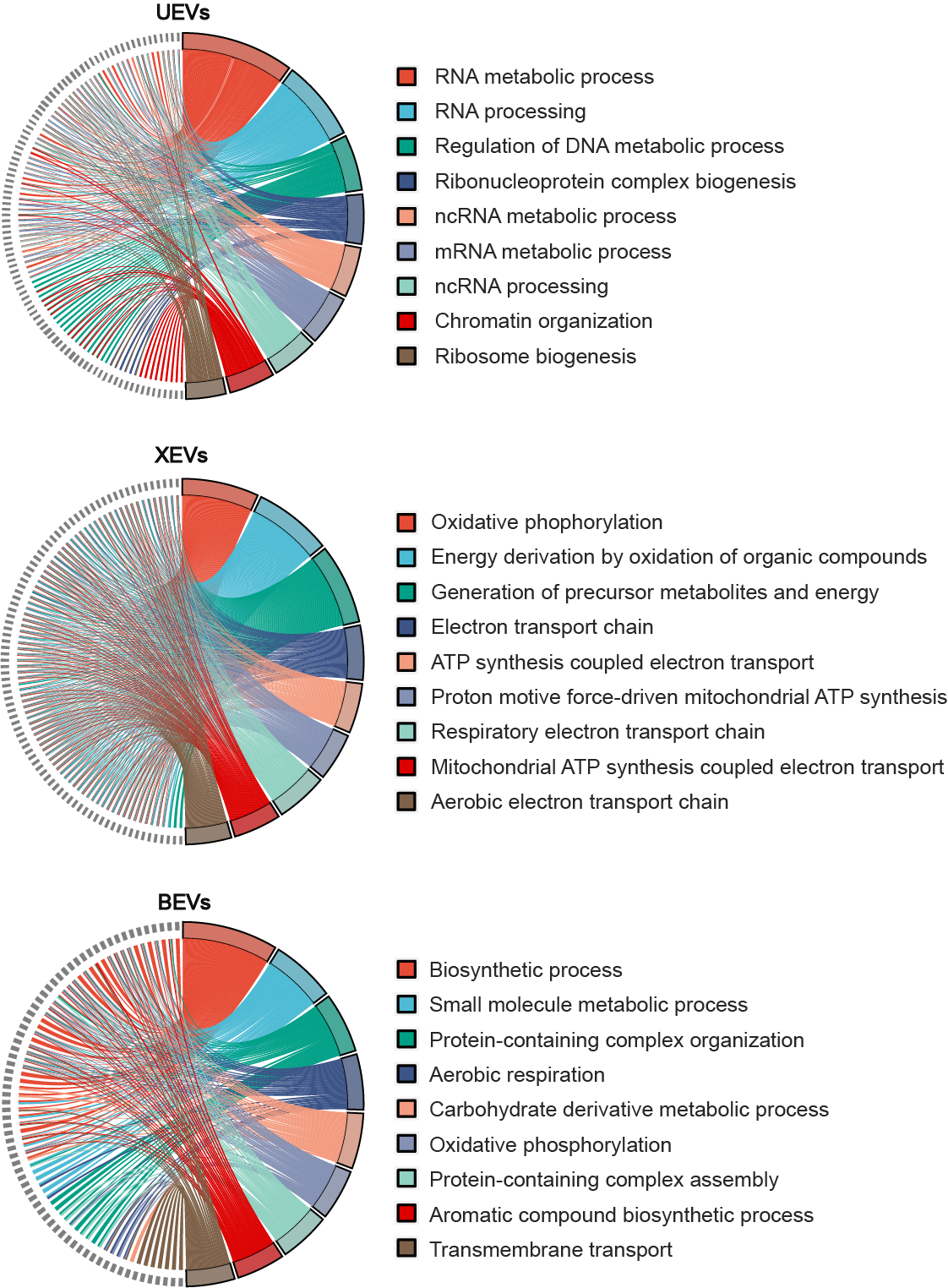


**Supplementary Figure 2** Classification of proteins of RT-EVs by biological process.


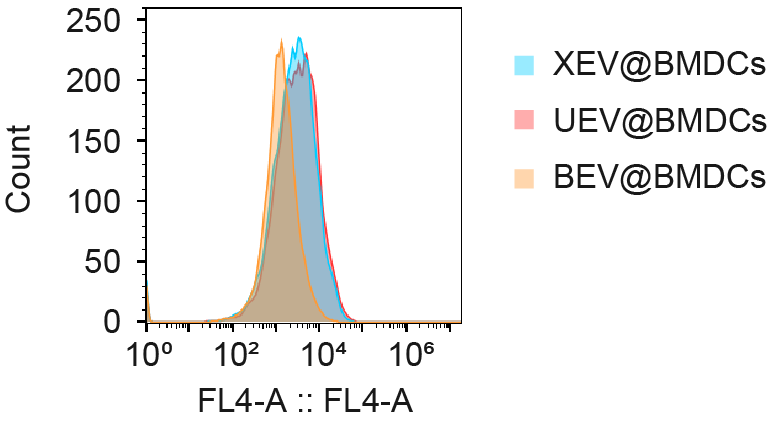


**Supplementary Figure 3.** Flow cytometric analysis of uptake level of DiR labeled UEVs, XEVs, and BEVs of BMDCs.


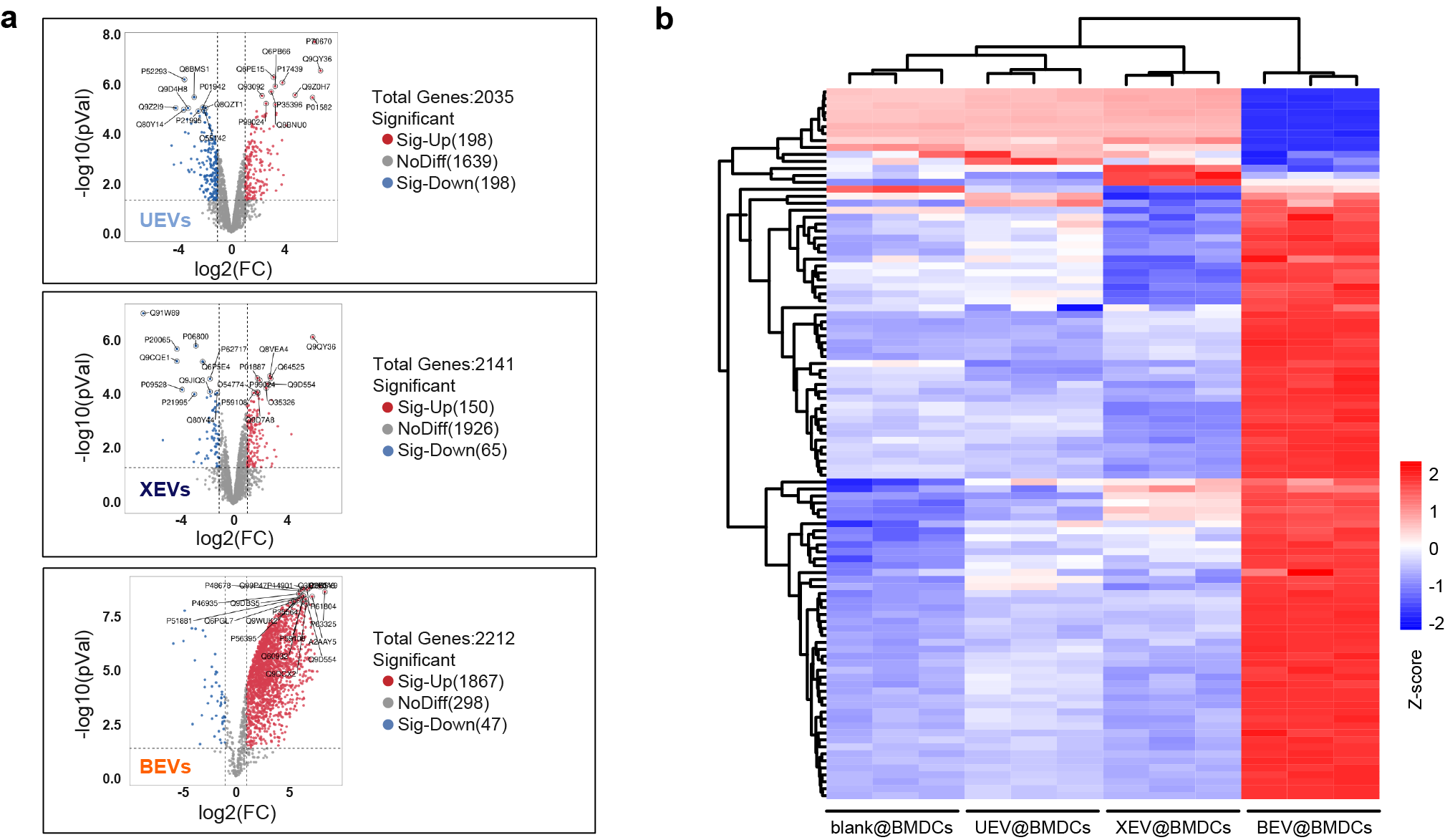


**Supplementary Figure 4.** **a**, Volcano plot of UEVs, XEVs, and BEVs pulsed BMDCs. Up-regulated and down-regulated proteins are displayed as red dots and blue dots respectively. **b**, Heat map displaying 299 disparate proteins in PBS, UEVs, XEVs and BEVs treated BMDCs, respectively.


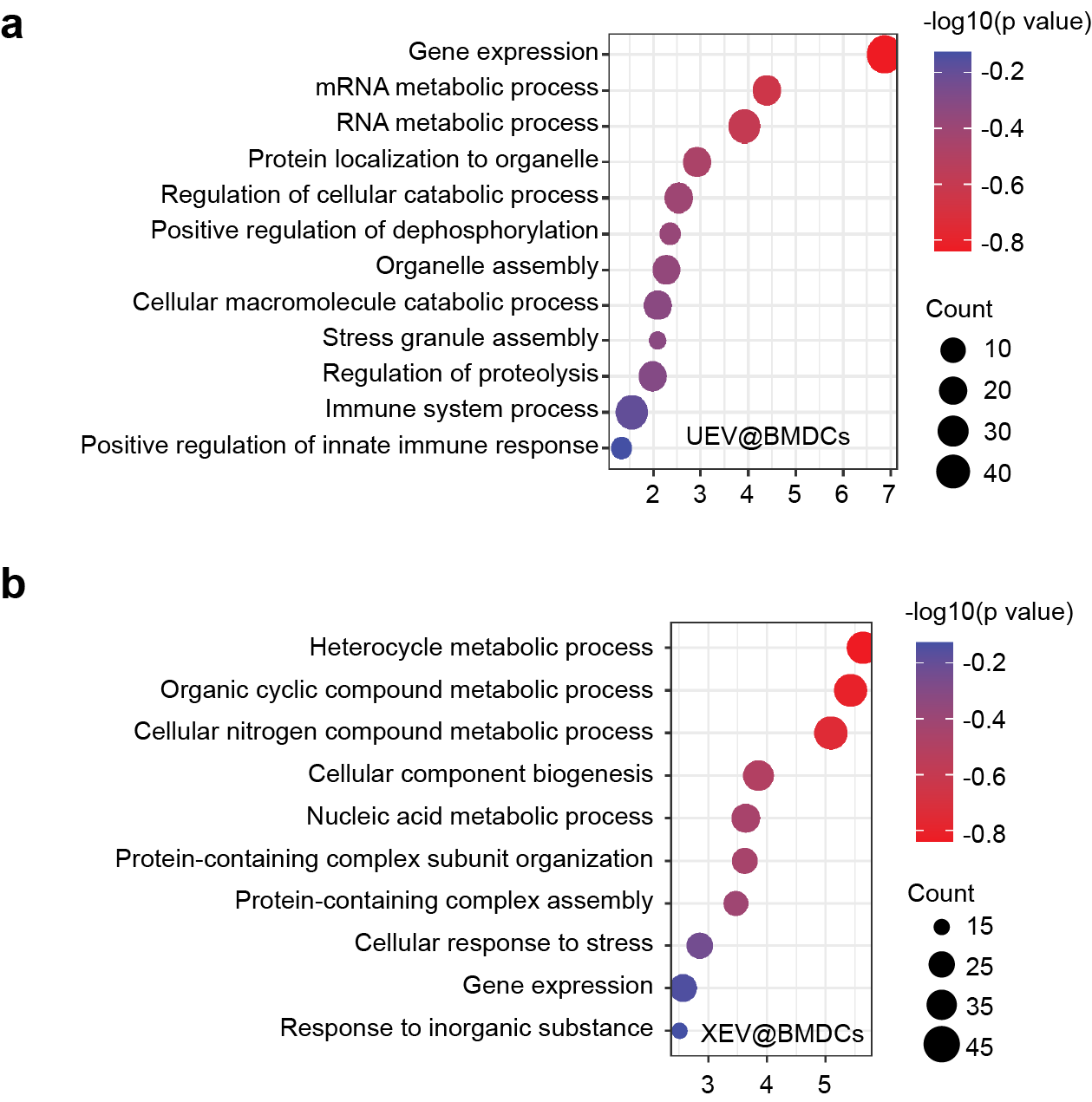


**Supplementary Figure 5.** Graphical representation of the enrichment of gene ontology terms of UEV@BMDCs and XEV@BMDCs group shown in **Fig 2b**.


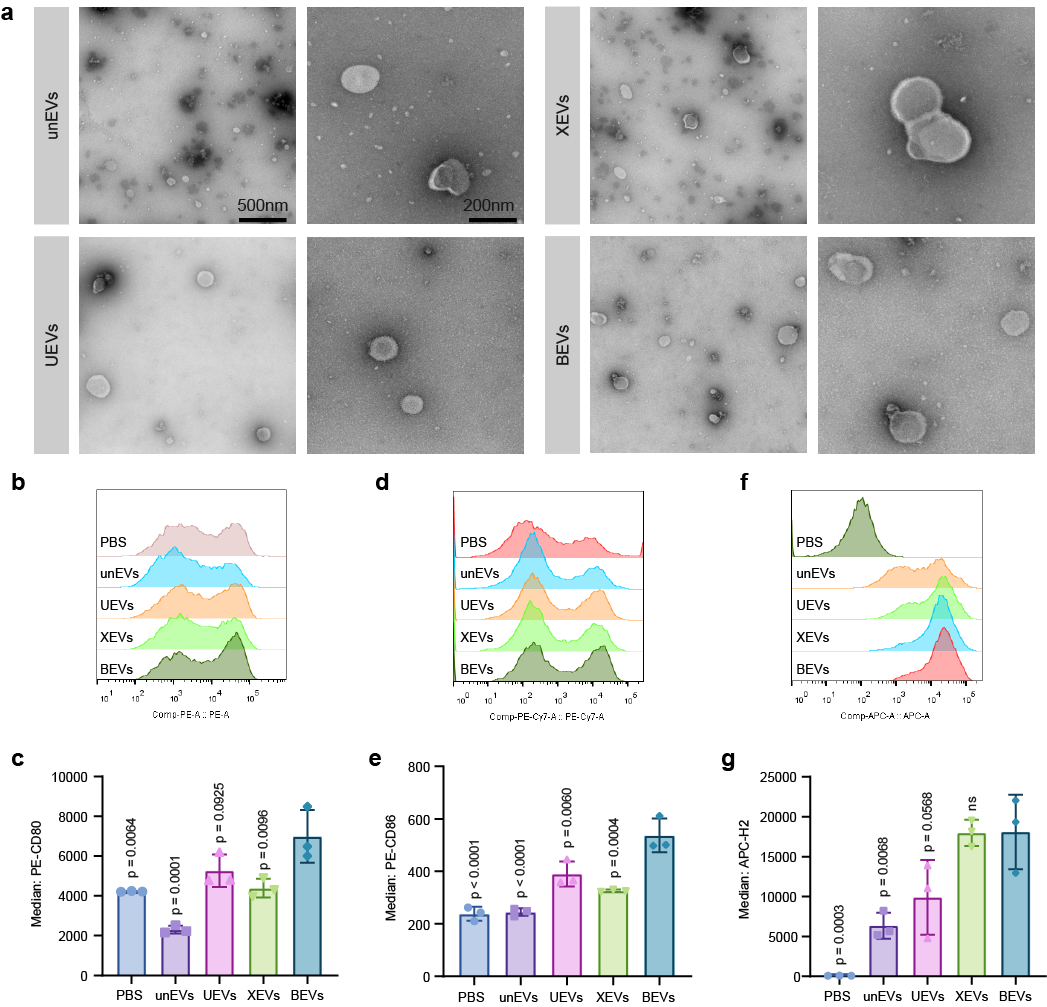


**Supplementary Figure 6.** **a**) Representative TEM images of unEVs, UEVs, XEVs, and BEVs. Histogram and column of PE-CD80 (**b** and **c**), PE/Cy7-CD86 (**d** and **e**), and APC-H2 (**f** and **g**) of EV-treated BMDCs. unEVs, untreated tumor cell-derived extracellular vesicles. Data presented as mean ± s.d. (n = 3 biologically independent samples).


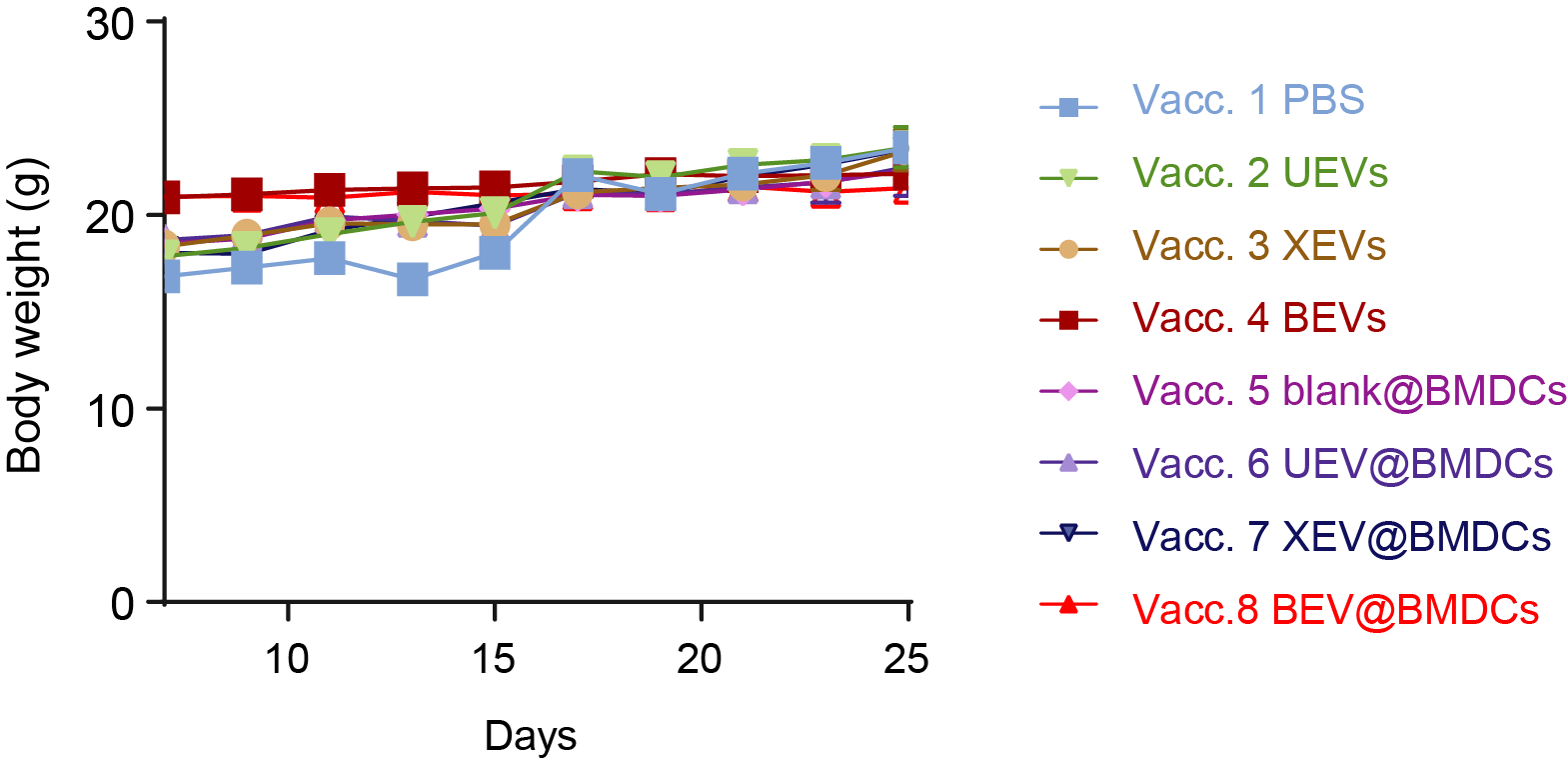


**Supplementary Figure 7.** Body weight of each immunized mice subcutaneously injected tumor cells was monitored during the experiment.


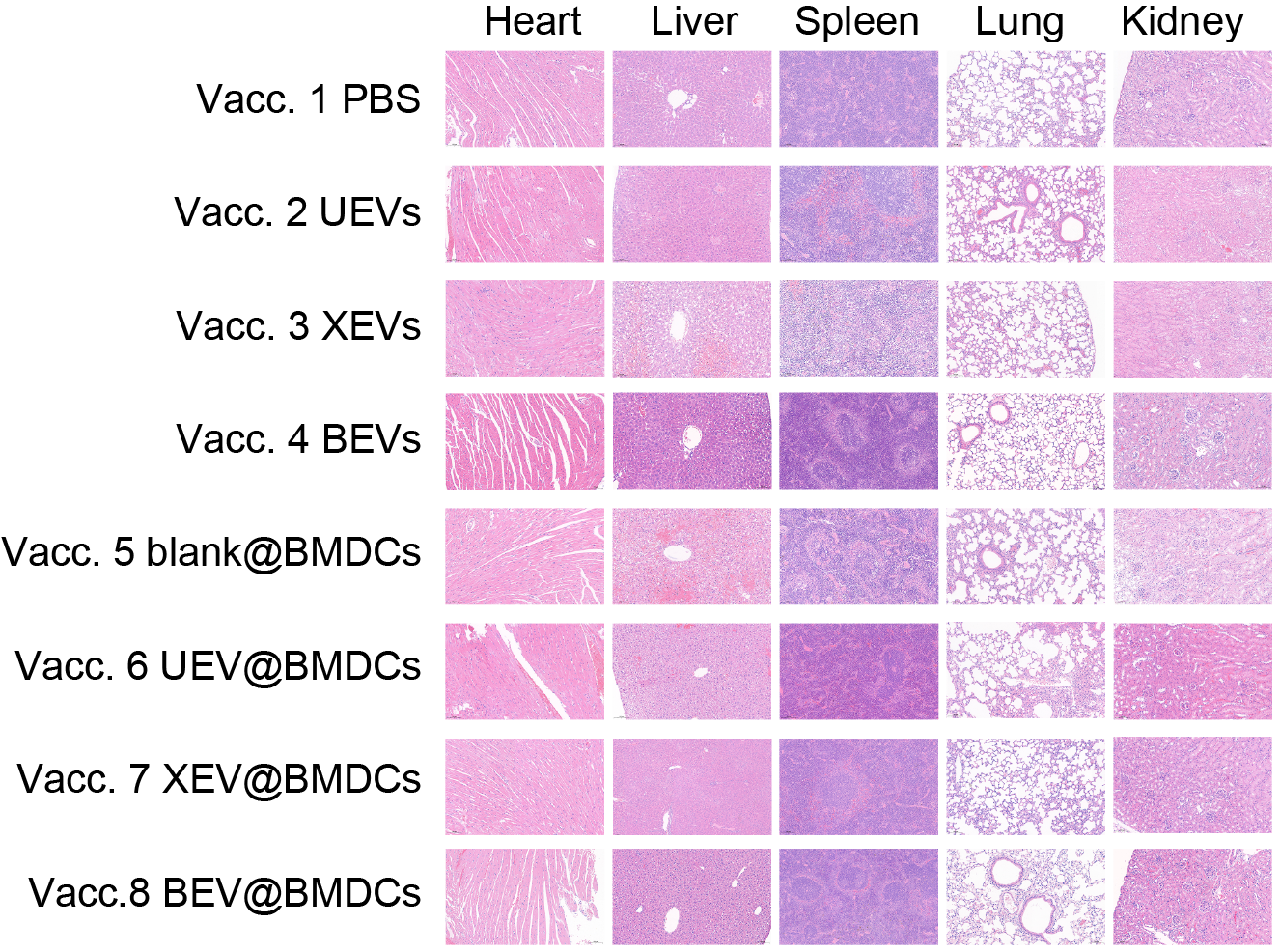


**Supplementary Figure 8.** H&E Histopathological observation of major organs (Heart, liver, spleen, lung and kidneys) from mice after treatments of different vaccinations (subcutaneous transplantation of B16-F10 tumor cells).


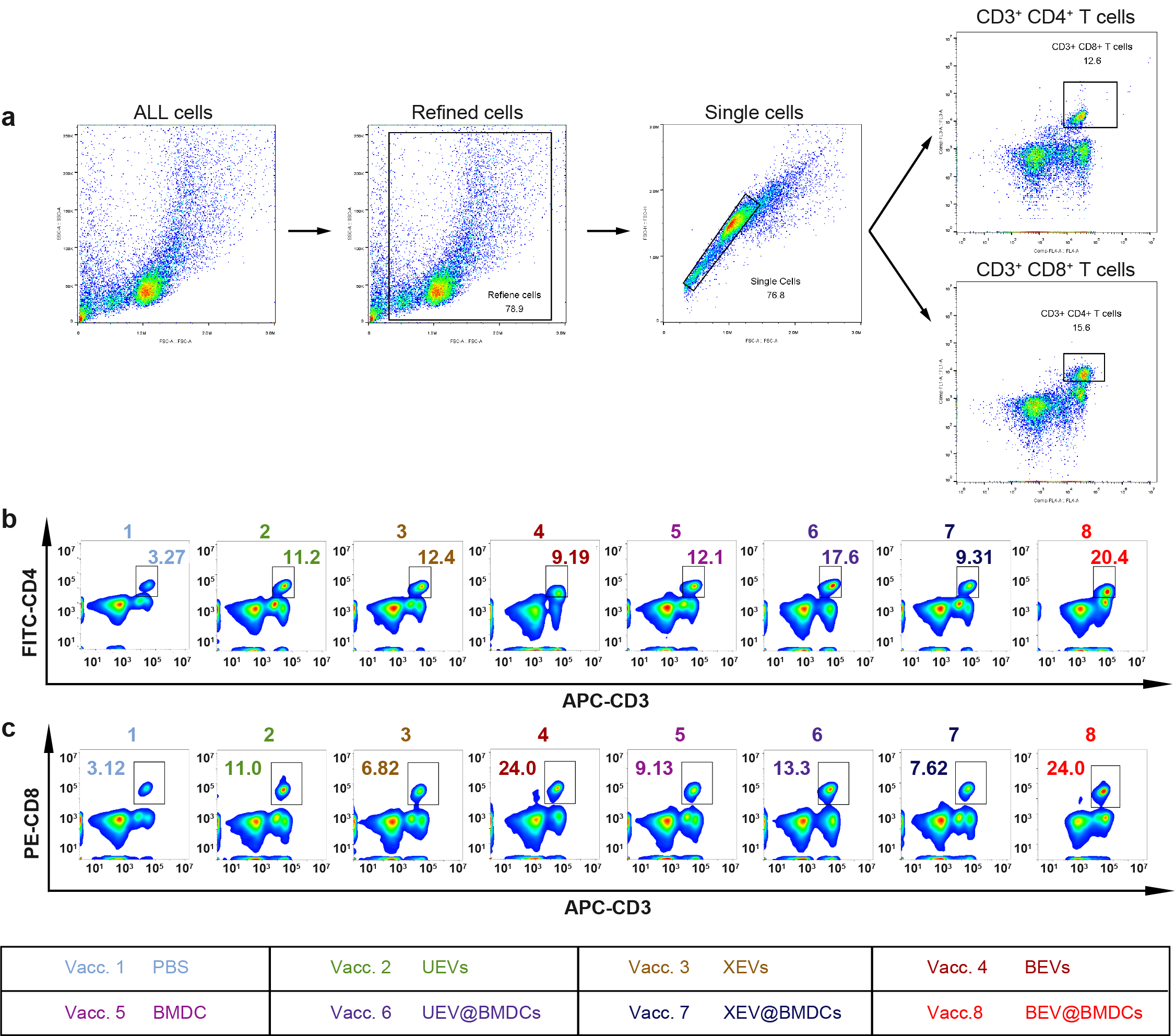


**Supplementary Figure 9.** **a**) The gating strategy of T cell population in flow cytometry analysis. Quantification of CD4^+^ T (**b**) or CD8^+^ T (**c**) cells in **spleens** of representative mice subcutaneously injected B16-F10 tumor cells.


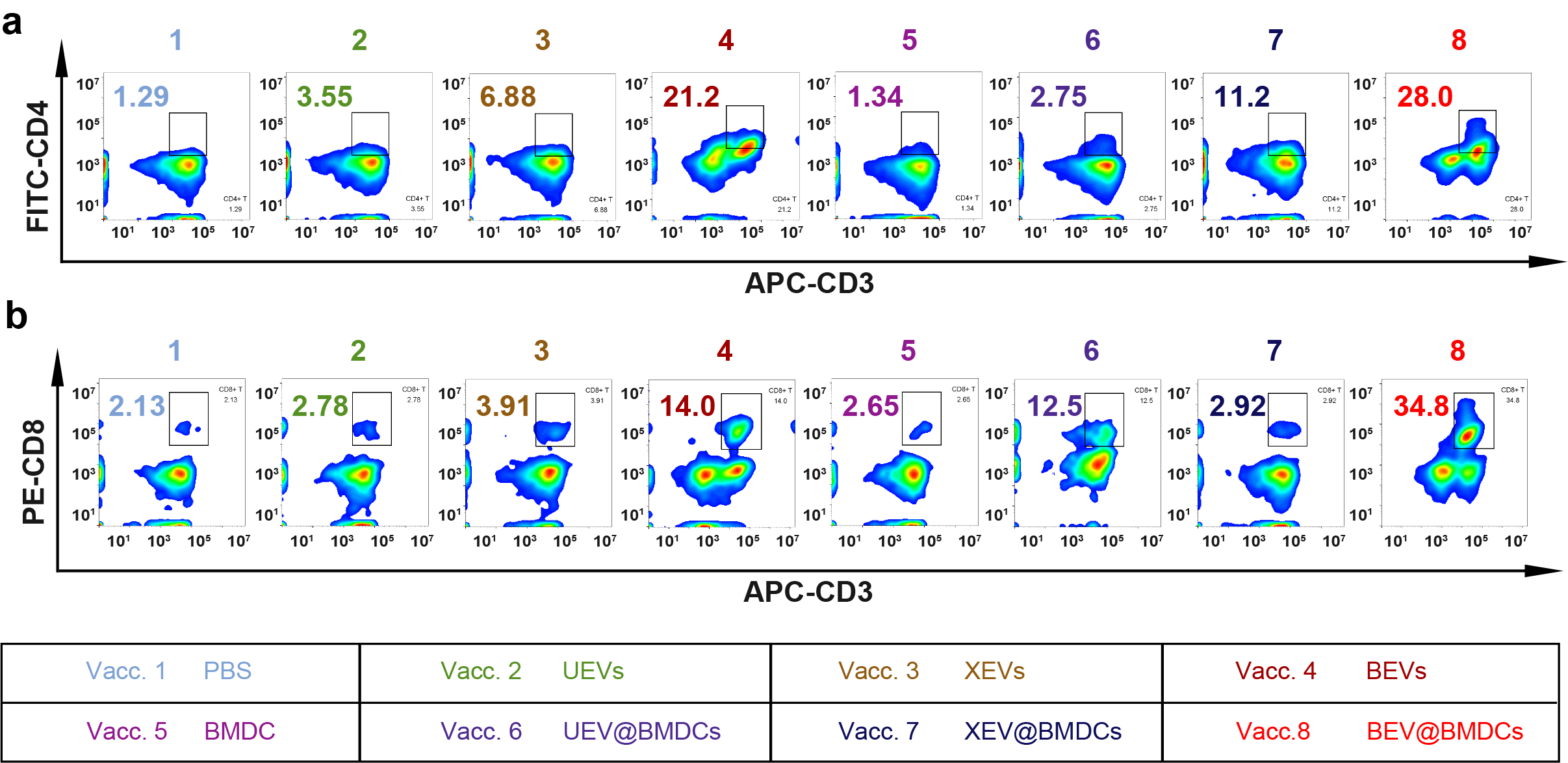


**Supplementary Figure 10.** Flow cytometry analysis and quantification of CD4^+^ T (**a**) or CD8^+^ T (**b**) cells in **tumors** of representative mice subcutaneously injected B16-F10 tumor cells.


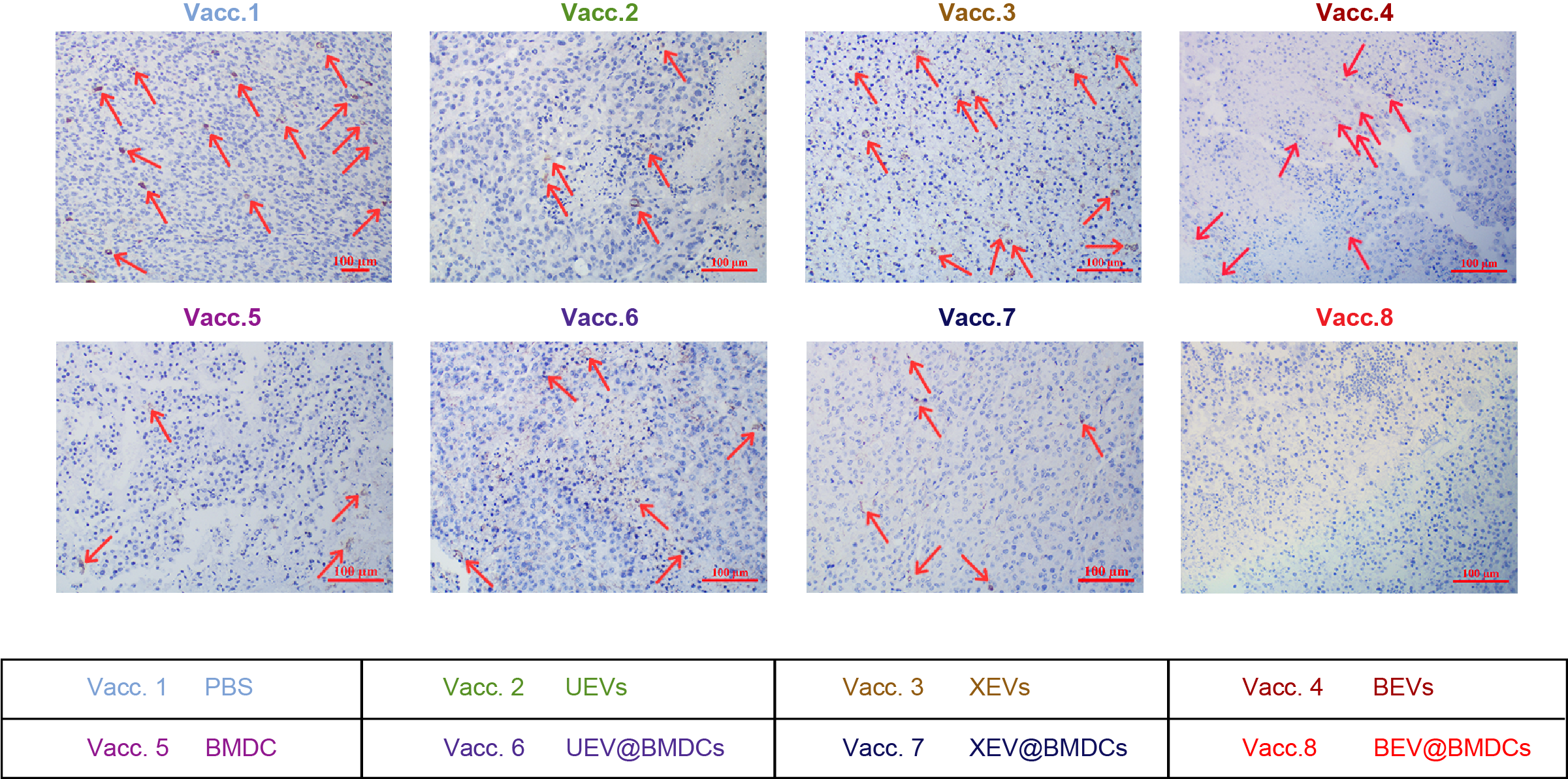


**Supplementary Figure 11.** Immunohistochemical staining of Treg cells (Foxp3 positive, brown staining) in the tumor tissues of mice after immunization with the according vaccines formulations. Scale bar, 100 µm.


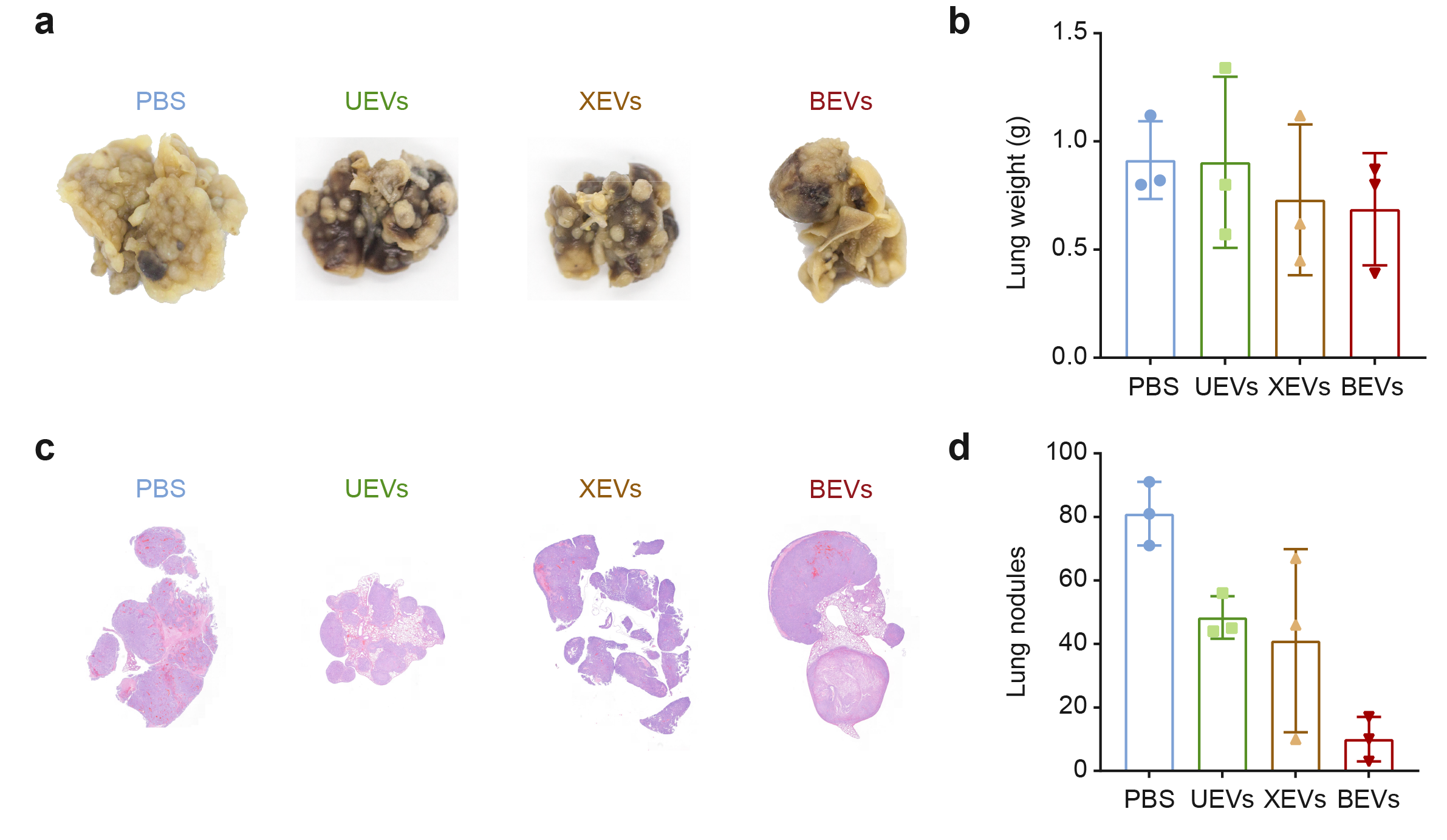


**Supplementary Figure 12.** **a**, Representative *ex vivo* images of the lung with metastatic foci from different groups. **b**, Lung mass measured after tumor challenge. **c**, H&E staining of the vaccinated mouse lungs collected after tumor inoculation. The nuclei-rich regions represent tumor metastases. **d**, The statistic result of the number of lung nodules. Data were shown as means ± s.d. (n=3) from 3 independent animals.


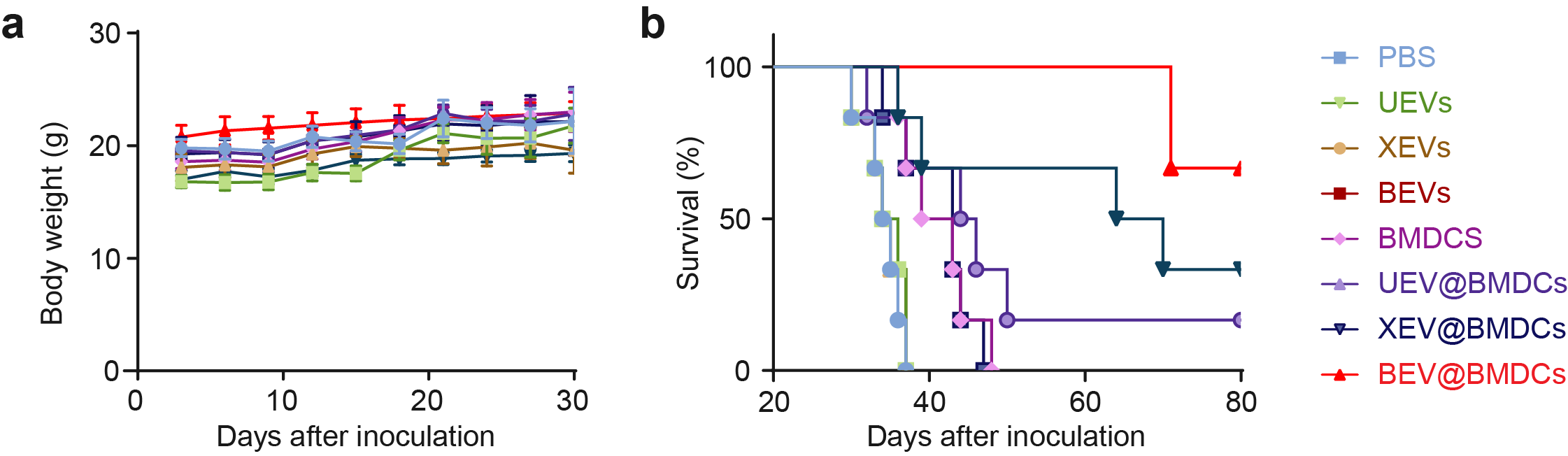


**Supplementary Figure 13.** **a)** Body weight of each tumor metastasis model was monitored. **b)** Kaplan-Meier survival curves of mice (metastasis models) in different treatment groups (n=6) from 6 independent animals.


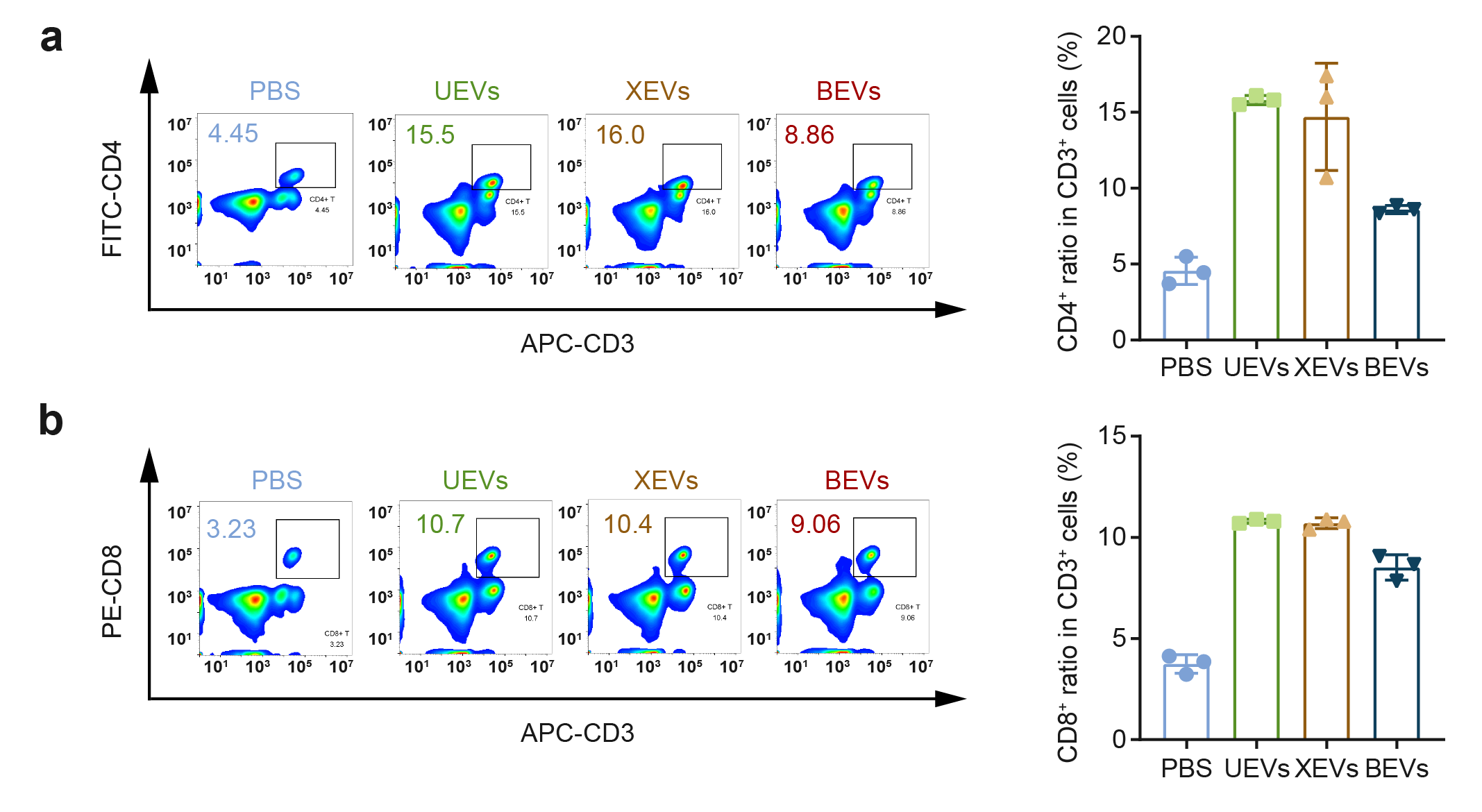


**Supplementary Figure 14.** Flow cytometry analysis and quantification of CD4^+^ T (**a**) or CD8^+^ T (**b**) cells in spleens of representative mice intravenously injected B16-F10 tumor cells.


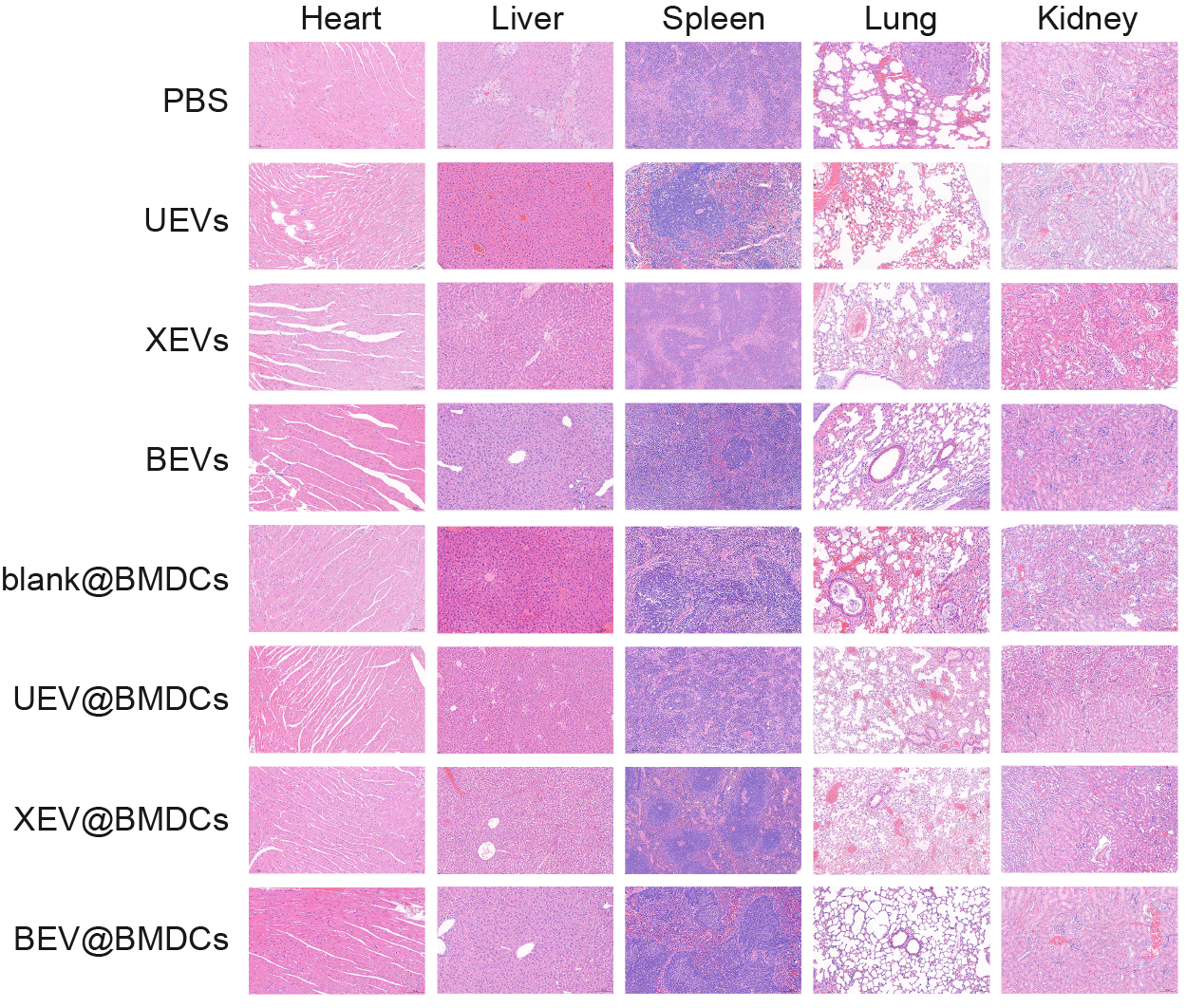


**Supplementary Figure 15.** H&E Histopathological observation of major organs (Heart, liver, spleen, lung and kidneys) from mice after treatments of different vaccinations (metastasis models).


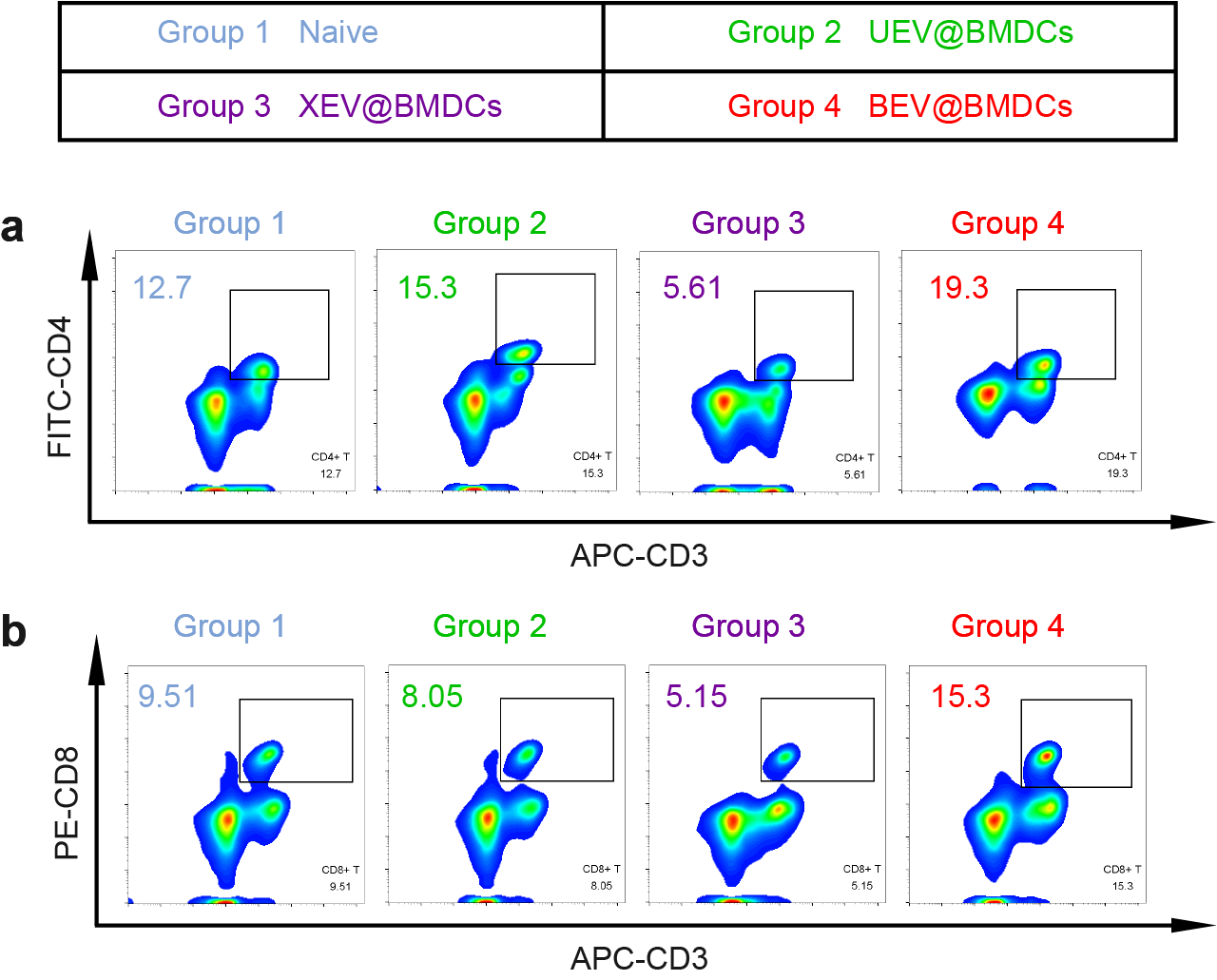


**Supplementary Figure 16.** Flow cytometry analysis and quantification of CD4^+^ T (**a**) or CD8^+^ T (**b**) cells in spleens of representative mice adopted splenocytes transfer.


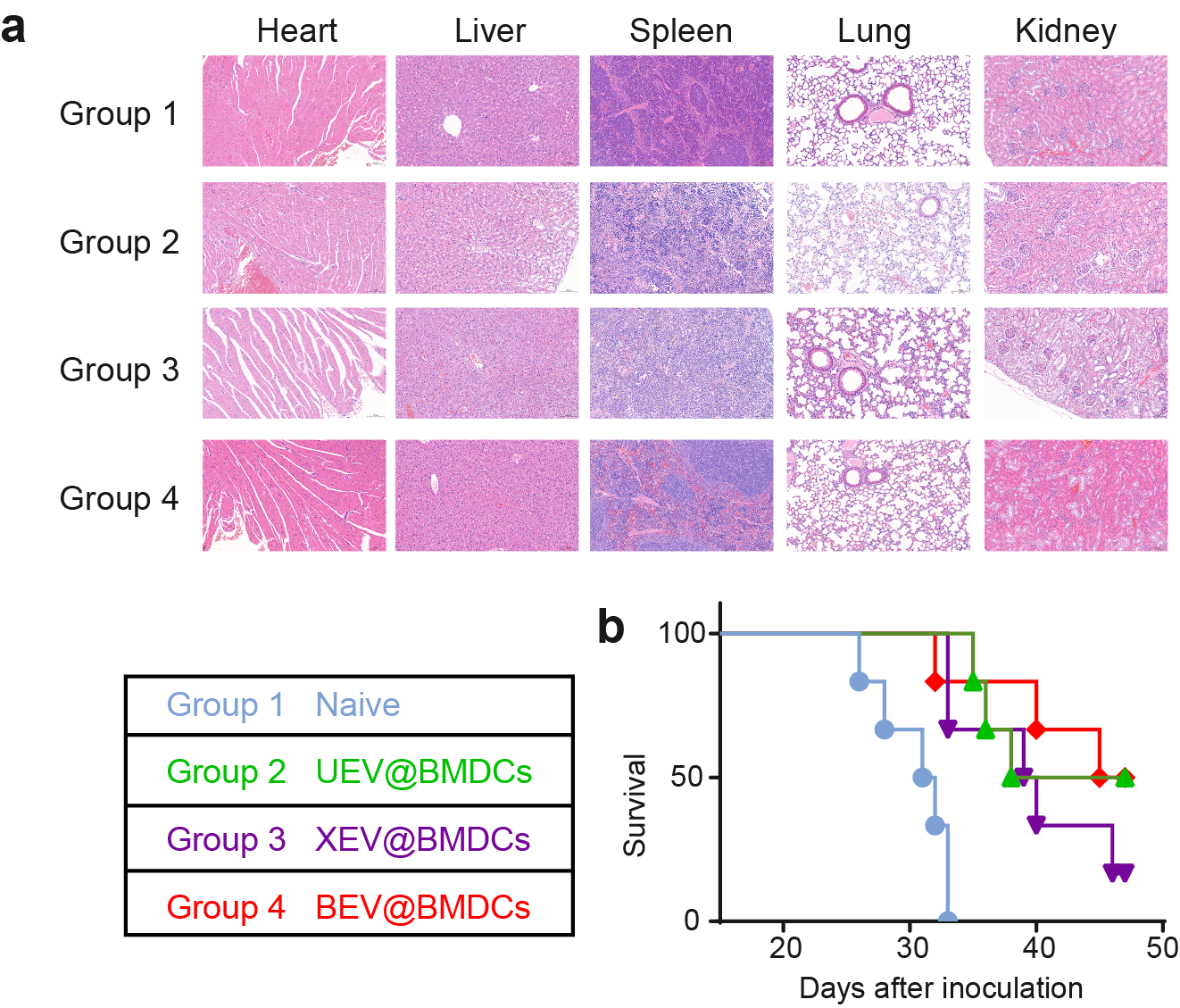


**Supplementary Figure 17.** **a**) H&E Histopathological observation of major organs (Heart, liver, spleen, lung and kidneys) from mice after treatments of different vaccinations (adoptive splenocytes transfer models). **b)** Kaplan-Meier survival curves of mice (adoptive models) in different treatment groups (n=6) from 6 independent animals.


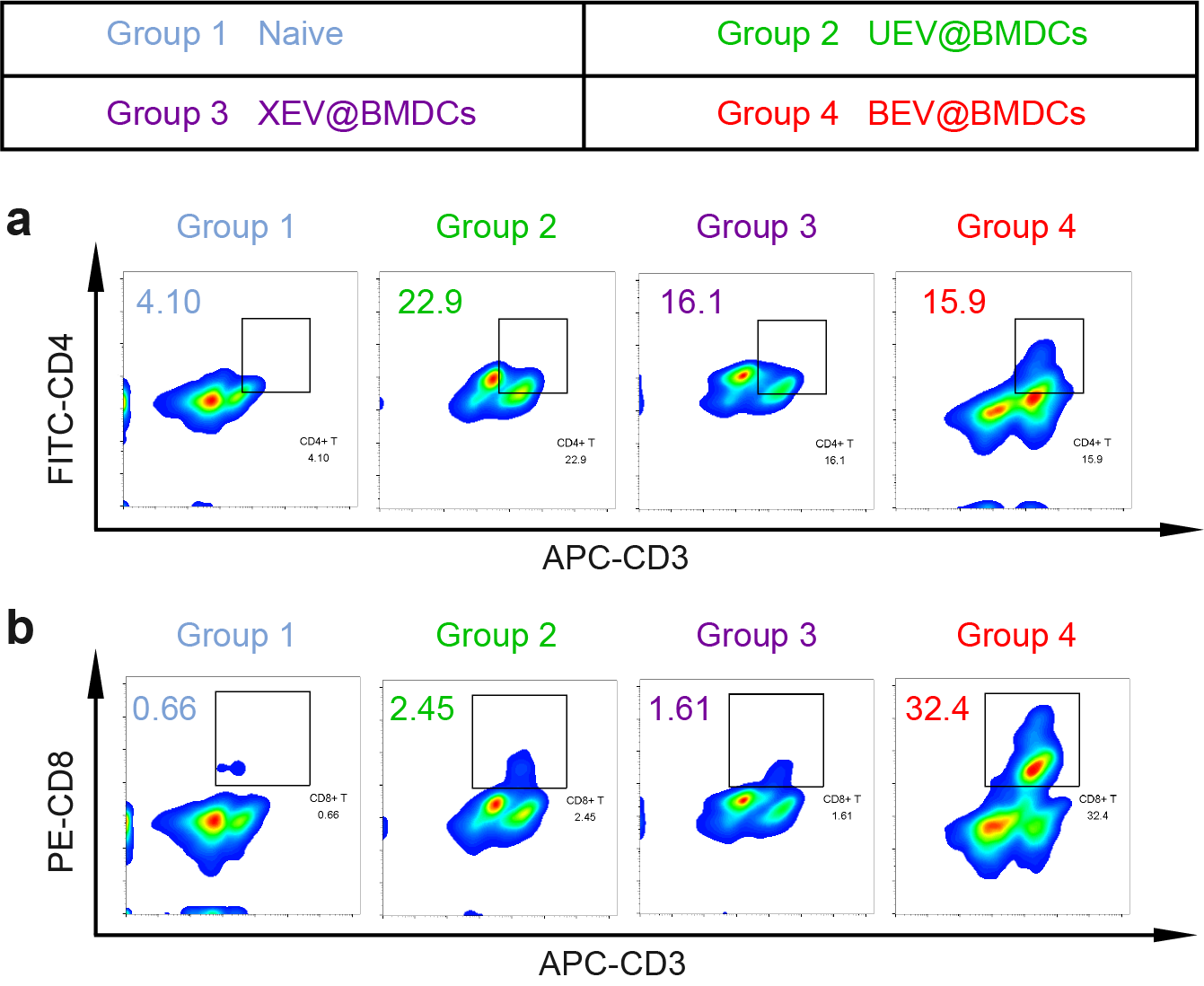


**Supplementary Figure 18.** Flow cytometry analysis and quantification of CD4^+^ T (**a**) or CD8^+^ T (**b**) cells in tumors of representative mice adopted splenocytes transfer.


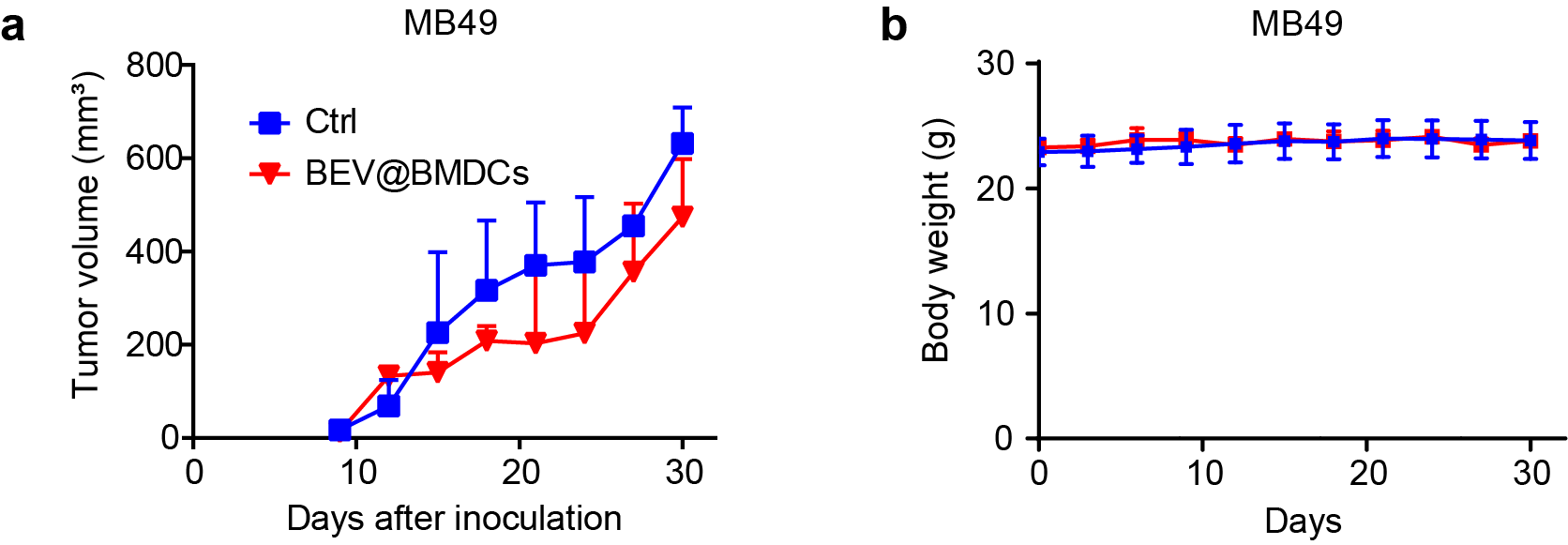


**Supplementary Figure 19.** Tumor growth (**a**) and body weight (**b**) of MB49 subcutaneous tumor model. Ctrl group represented mice were transferred by splenocytes from naïve mice. BEV@BMDCs group represented mice were transferred by splenocytes from survived mice treated by BEV@BMDCs.


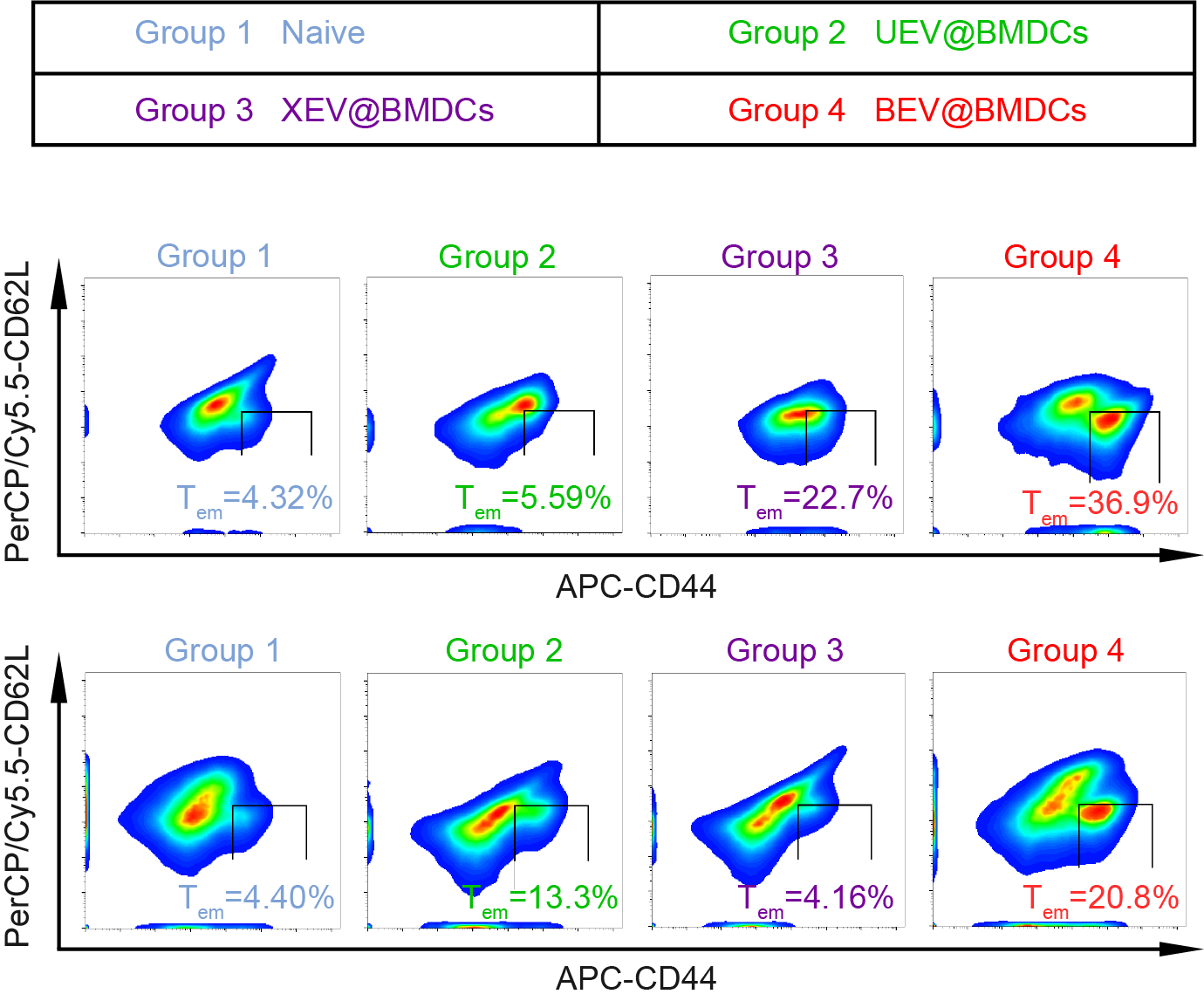


**Supplementary Figure 20.** **a,** Proportions of CD44^＋^CD62L^－^ cells in CD4^+^ T cells of tumor tissues of adoptive models. **b,** Proportions of CD44^＋^CD62L^-^ cells in CD8^+^ T cells of tumor tissues of adoptive models.


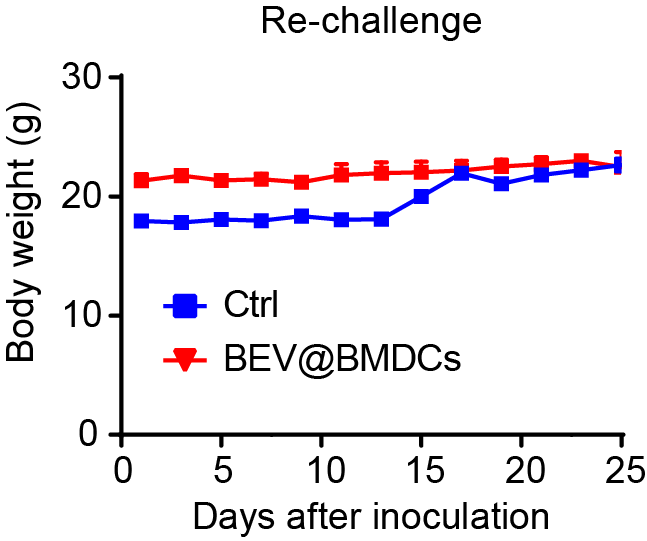


**Supplementary Figure 21.** Body weight of re-challenge models.
